# Reductive stability evaluation of 6-azopurine photoswitches for the regulation of CKIα activity and circadian rhythms†

**DOI:** 10.1039/d1ob00014d

**Published:** 2021-02-19

**Authors:** Dušan Kolarski, Akiko Sugiyama, Theo Rodat, Albert Schulte, Christian Peifer, Kenichiro Itami, Tsuyoshi Hirota, Ben L. Feringa, Wiktor Szymanski

**Affiliations:** a Stratingh Institute for Chemistry, University of Groningen Nijenborgh 4 9747 AG Groningen The Netherlands b.l.feringa@rug.nl; b Institute of Transformative Bio-Molecules (WPI-ITbM), Nagoya University Furo-cho Chikusa Nagoya 464-8601 Japan thirota@itbm.nagoya-u.ac.jp; c Department of Pharmaceutical and Medicinal Chemistry, Christian-Albrechts-University of Kiel Gutenbergstraße 76 24118 Kiel Germany; d Medical Imaging Center, University Medical Center Groningen, University of Groningen Hanzeplein 1 9713 GZ Groningen The Netherlands w.szymanski@umcg.nl

## Abstract

Photopharmacology develops bioactive compounds whose pharmacological potency can be regulated by light. The concept relies on the introduction of molecular photoswitches, such as azobenzenes, into the structure of bioactive compounds, such as known enzyme inhibitors. Until now, the development of photocontrolled protein kinase inhibitors proved to be challenging for photopharmacology. Here, we describe a new class of heterocyclic azobenzenes based on the longdaysin scaffold, which were designed to photo-modulate the activity of casein kinase Iα (CKIα) in the context of photo-regulation of circadian rhythms. Evaluation of a set of photoswitchable longdaysin derivatives allowed for better insight into the relationship between substituents and thermal stability of the *cis*-isomer. Furthermore, our studies on the chemical stability of the azo group in this type of heterocyclic azobenzenes showed that they undergo a fast reduction to the corresponding hydrazines in the presence of different reducing agents. Finally, we attempted light-dependent modulation of CKIα activity together with the accompanying modulation of cellular circadian rhythms in which CKIα is directly involved. Detailed structure–activity relationship (SAR) analysis revealed a new potent reduced azopurine with a circadian period lengthening effect more pronounced than that of its parent molecule, longdaysin. Altogether, the results presented here highlight the challenges in the development of light-controlled kinase inhibitors for the photomodulation of circadian rhythms and reveal key stability issues for using the emerging class of heteroaryl azobenzenes in biological applications.

## Abstract 2

6-Azopurines were evaluated for their reductive stability, and the ability to modulate CKIα activity and cellular circadian rhythms, revealing key challenges for long-term activity modulation utilizing chronophotopharmacology.

## Graphical abstract

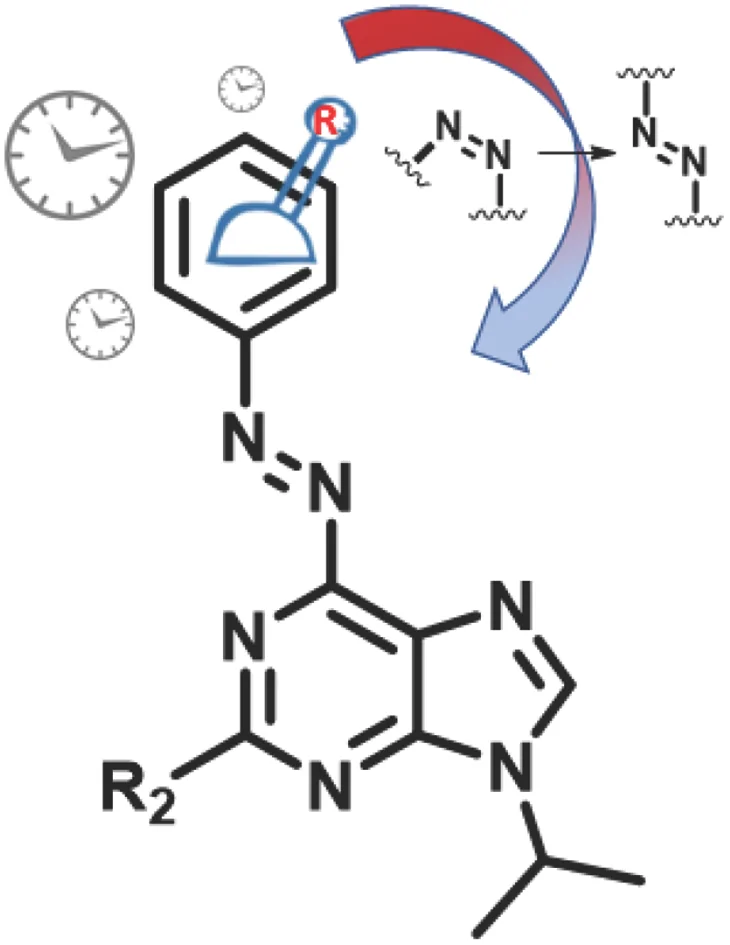

## Introduction

Protein kinases constitute a major target class for drug development.^1^ The human kinome consists of ∼500 kinases with various key cellular functions.^2^ Due to this vastness of the kinome, the general similarities in the ATP-binding domain and a uniform catalytic mechanism, the development of specific kinase- or kinase type-selective inhibitors remains challenging.^3^ This limitation could be partially overcome by considering, besides the biochemical target selectivity, the site specificity – *e.g.* by limiting not the enzymes with which the compound interacts, but the parts of organs of the body in which the compound is active, thereby preventing unwanted off-site activities in the future.

Such external control over inhibitor potency in time and space could be enabled using light to locally and reversibly activate the bioactive compound only at the desired site of action. This concept forms the basis for the photopharmacological approach: photopharmacology is an emerging field of chemical biology that utilizes photo-responsive molecules to enable control over the activity of a drug, using light.^4–7^ The main tools of photopharmacology are molecular photoswitches,^8^ which can be reversibly switched between two or more isomeric forms using light of different wavelengths. Such control has been established over bacterial communication and resistance buildup,^9,10^ ion channels,^11^ G protein-coupled receptors,^12^ lipid membranes,^13^ and nucleic acids.^14,15^ However, up to this point, the photocontrol of kinase activity has proven to be highly challenging for photopharmacology, leading often to chemically unstable compounds, undesired photochemical side-reactions or photoswitchable kinase inhibitors that show little difference in potency in their photoisomeric states. Recent examples of photoswitchable kinase inhibitors include photomodulation of RET,^16^ PKC,^17^ MEK1,^18^ VEGFR2^19^ and BRAF^V600E^ (ref. 20) enzymes.

Here we report the evaluation of a library of green-light switchable 6-azopurines for the photocontrolled inhibition of casein kinase Iα (CKIα) in the context of our efforts towards the light regulation of circadian rhythms. Circadian rhythms are based on endogenous biochemical oscillations with a ∼24 h cycle observed in almost every cell in the human body.^21,22^ Various studies have linked the disruption of these rhythms to a wide variety of diseases and disorders.^23–25^ In mammals, at the molecular level, circadian oscillations are driven by negative feedback loops of clock genes. Casein kinase I (CKI, isoforms δ, ε, and α) family proteins play a key role in these loops (Fig. 1A) by phosphorylating the PER protein and thus promoting its proteasomal degradation and maintaining the circadian rhythms on the ∼24 h base. Control of circadian cycles by employing small molecules has recently been investigated, opening fascinating opportunities in disease control.^26–29^ High-throughput screening supported by rational synthetic design led to the discovery of small-molecule modulators of the circadian period, mostly showing period lengthening,^30–36^ but some also exhibiting period shortening effect.^37–39^ The Kay group identified a potent modulator of cellular circadian rhythms, dubbed ‘longdaysin’ (Fig. 1B), which showed IC_50_ values of 8.8 and 5.6 μM for CKIα and CKIδ, respectively.^30^ However, since the core clock mechanism resides throughout the mammalian body,^22,40^ circadian period modifiers, such as longdaysin, display a non-selective period modulation of all peripheral clocks as well as of the master clock, suprachiasmatic nucleus (SCN). Establishing photocontrol over their activity might enable site-selective activation and pave the way towards chronophotopharmacology.

**Fig. 1:**
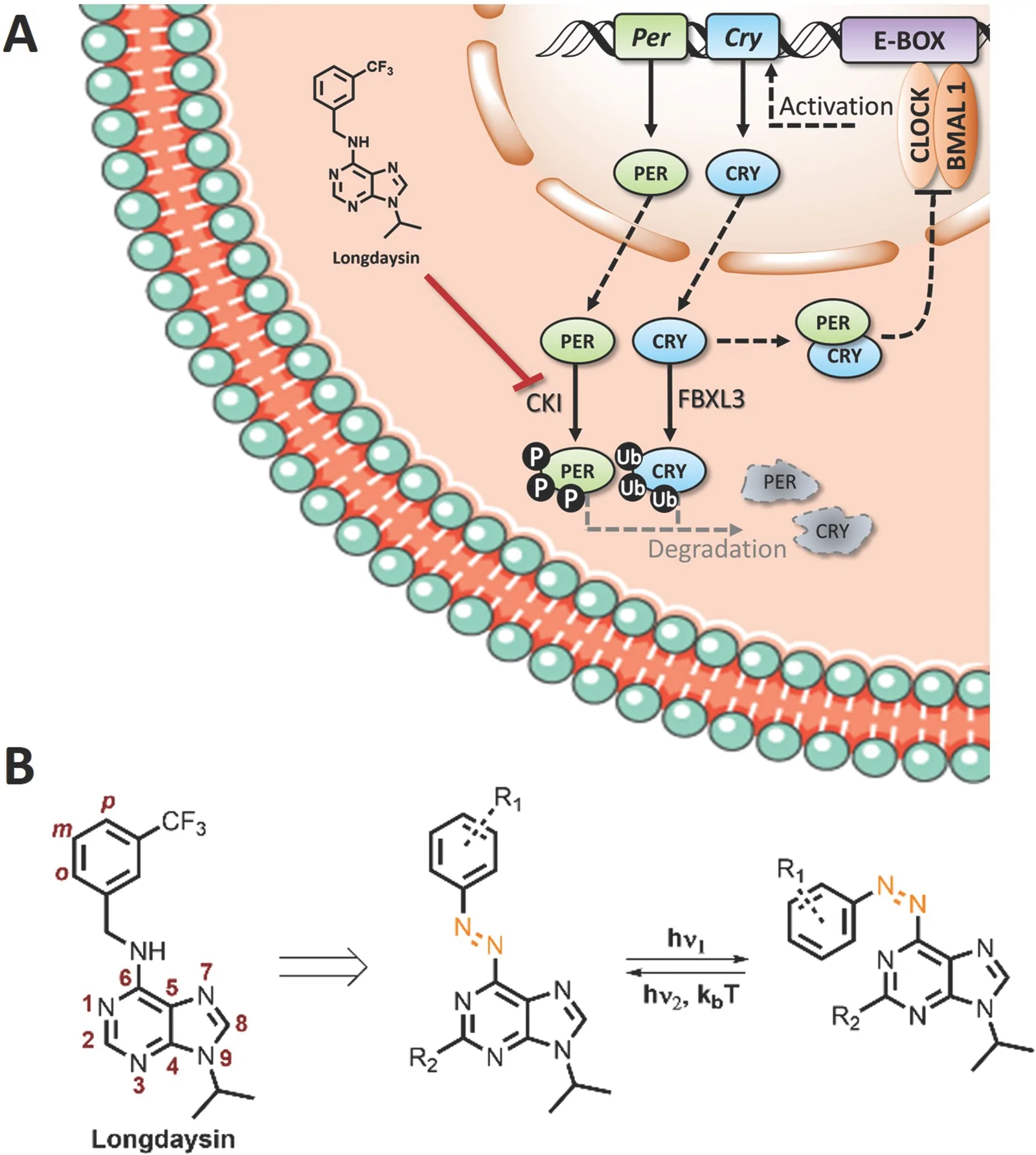
(A) Schematic representation of the circadian feedback loop and its modulation with longdaysin. (B) Structure of longdaysin and photo-isomerization of its azo-analogue.

Towards enabling the photocontrol by switchable circadian rhythm regulators, we have recognized the *N*-benzyl-arylamine scaffold of longdaysin as the candidate for the azologization approach,^41^ in which azobenzene photoswitches are introduced into the structure of bioactive compounds with only a minimal overall structural change. Azobenzenes are a highly versatile and widely applied class of photoswitches^42,43^ that undergo light-induced isomerization between the thermally-stable *trans*-isomer and the unstable *cis*-isomer (Fig. 1B). They have been widely exploited in photopharmacology as the most versatile and well-understood photocontrolled tools.^5,6^ Especially the recent interest in heterocyclic azobenzenes is inspired by the increasing number of methods for their synthesis^5,14,44^ and the common presence of heterocyclic moieties in drugs.^45–47^ Despite the fact that SAR analysis of longdaysin was conducted by extensive structural modifications of its C(2) and N(9) positions,^35^ to the best of our knowledge, modification of the benzene ring (*o*, *m*, *p*, Fig. 2) has never been investigated. Thus, a synthetic methodology and a library of photoswitchable compounds developed previously by our group^48^ were evaluated for enabling reversible circadian clock modulation (Fig. 1).

**Fig. 2:**
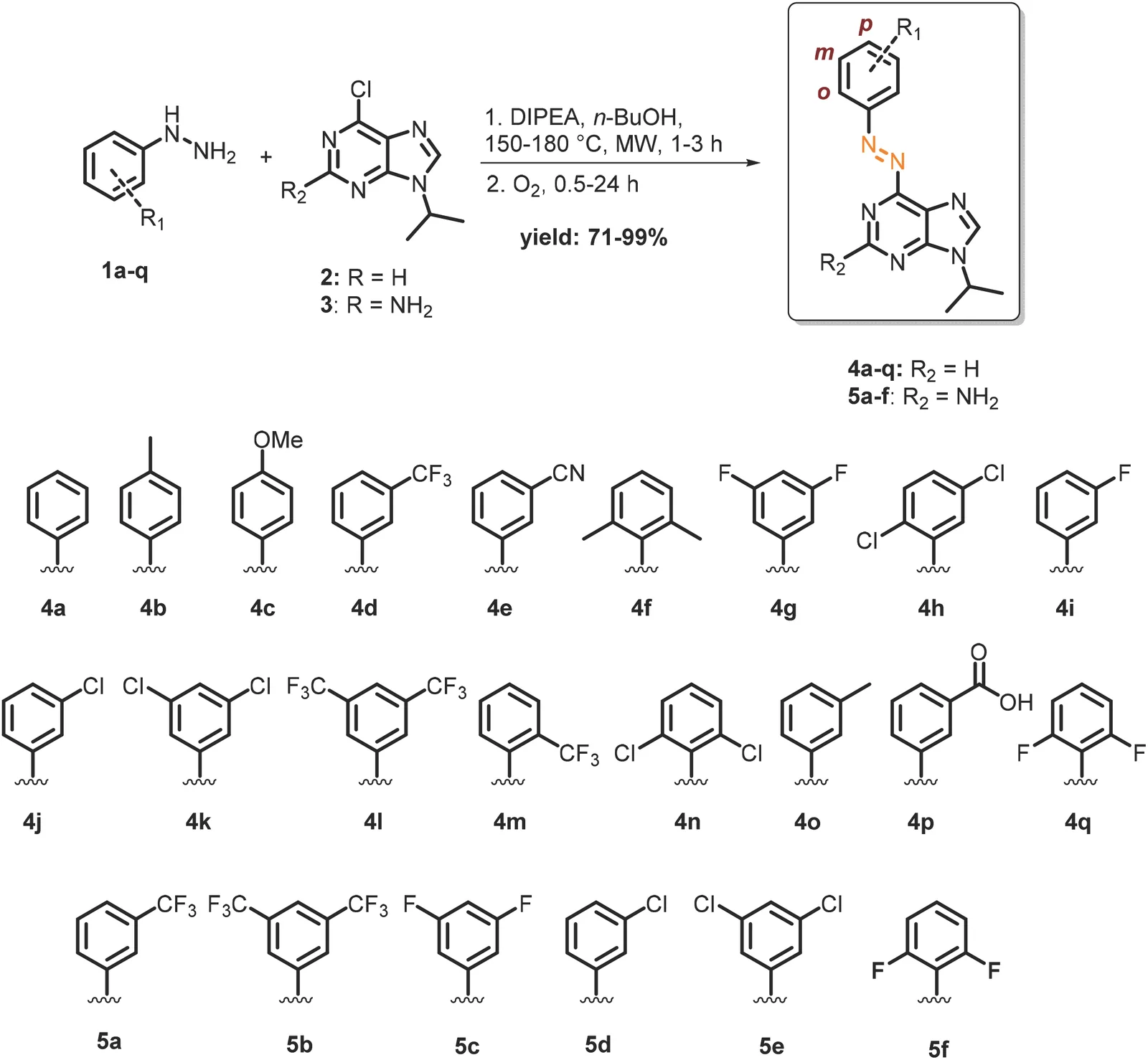
Synthesis of longdaysin azologs 4a–q and 5a–f.

## Results and discussion

Microwave-assisted nucleophilic aromatic substitution of 9-chloro purines (2 and 3) with substituted aryl hydrazines (1a–q), followed by oxidation (Fig. 2), provided in good to high yields the desired photoswitches based on the purine core (R_2_ = H, 4a–q; R_2_ = NH_2_, 5a–f). The choice of substituents was primarily driven by investigation of their influence on the biological activity, but was also aimed at obtaining the optimal photochemical properties, such as visible-light photo-isomerization and longer thermal half-lives of the metastable *cis*-isomers. Since the ultimate application of this work is to control the biological activity, substituents were chosen to electronically resemble the electron-withdrawing CF_3_ group present originally in longdaysin. These substituents ranged from strongly electron-withdrawing to weakly electron-donating groups, with the exception of compound 4c, the only one with a strong electron-donating methoxy substituent.

For the application of azobenzenes in biological systems, their photochemical properties must be adjusted for a particular purpose. The most common parameters to be optimized are the photostationary state distribution (PSD) of isomers under irradiation with light of different wavelengths, thermal half-life of the metastable isomer, wavelength used for photo-isomerization, and (photo)chemical stability. Given that the cellular circadian assay takes several days, thermal *cis*-to-*trans* isomerization had to be minimized to permit a pronounced biological effect of the *cis*-isomer, with the least background activity of the *trans*-isomer. Thus, by modifying the benzene ring, next to increased biological activity (*vide infra*), we also aimed for longer half-lives. If this condition is not met, continuous or pulse irradiation with cell-nontoxic light (>500 nm)^49–51^ would be required during the measurement to keep the content of the *cis*-isomer as high as possible for a prolonged period. However, this is not possible in circadian rhythm assays due to the nature of the experimental setup in which the luminescence of the cells is being measured in a plate reader under carefully maintained (dark) conditions, precluding continuous or pulsed irradiation.^37^

### Photo-isomerization

The wavelength used for isomerization is one of the key features for utilization of azobenzene in biological systems.^51,52^ Generally, UV-light is needed for *trans*-to-*cis* isomerization, while back-isomerization can be achieved by either thermal relaxation or visible light irradiation.^43^ As UV-light is strongly absorbed and scattered by the skin, visible-light mediated photo-isomerization is highly desirable due to increased tissue penetration and reduced phototoxicity.^53,54^ Heterocyclic azobenzenes, presented in this work, allow for using green light (*λ*_max_ = 530 nm) for *trans*-to-*cis* photo-isomerization (Fig. S4†). To quantitatively determine *trans*-to-*cis* conversion with green light, PSDs of two switches (4f and 5e) were measured by ^1^H-NMR (DMSO-*d*_6_, 1 mg mL^−1^, 25 °C, Fig. S4†). The photostationary state was achieved after 2 h of irradiation with green light. For azopurine 4f, the PSS distribution (*cis* :* trans*) was determined to be 61 : 39 while for 5e it was 71 : 29.

### Thermal half-life of compounds *cis*-4a–q and *cis*-5a–f

After the photostationary state was reached, thermal back-isomerization was studied in DMSO, CKI assay buffer and the medium used in the cellular assay (Table 1). The relationship between the structure and thermal stability in DMSO revealed that back-isomerization was highly dependent on the electronic nature of the substituents, as well as their position on the aromatic ring. Increased electron density of the benzene ring created a push–pull system with an electron-poor adenine core, causing shorter half-lives.^55^ Thus, *para*-methoxy substituted azopurine 4c exhibited the shortest half-life of 17 s. On the other hand, increasing the electron density of the purine core by incorporation of the C(2)-amino group (5a–f) had the opposite effect. Further analysis has shown that *para*- and *meta*-substituents on the benzene ring had a very limited influence on the thermal stability of the *cis*-isomer. Conversely, incorporation of *ortho*-substituents had a significant impact on the half-lives. Interestingly, the half-life of di-*ortho*-methyl compound 4f was shorter than of mono-*ortho*-trifluoromethyl compound 4m, indicating the importance of not only the steric effects but also the electronic effects of the *ortho*-substituents. Inspired by the work of Woolley^51,52^ and Hecht^56^ and our recent studies,^57^ we expected to enhance the thermal stability of the *cis*-isomer by introducing *ortho* fluoro- or chloro-substituents. Incorporation of only one chlorine atom (4h) led to a significantly longer half-life of >1 h. The thermal half-life for the relaxation of di-*ortho*-fluoro compound 4q was 1.4 h, while di-*ortho*-chloro compound 4n displayed the longest half-life among 2-H-azopurines of almost 3 h. Remarkably, di-*ortho*-fluoro compound 5f exhibited the slowest thermal relaxation, with a half-life of almost 12 h. In summary, for longdaysin azologs 4 and 5, a very broad range of half-lives in DMSO (from 17 s to 12 h) were obtained. Altogether, the stability of the *cis*-isomer could be fine-tuned by controlling the electronic and steric effects of the substituents.

**Table 1:** Thermal *cis*-to-*trans* isomerization in three different media: DMSO (40 μM, 25 °C), CKI assay buffer (40 μM, 30 °C) containing ∼100 μM dithiothreitol (DTT) and cellular assay medium (40 μM, 35 °C). Half-life of the *trans*-azobenzene (40 μM) reduction in CKI buffer containing 500 μM DTT (30 °C)

Compound	*t*_1/2_, *cis*-to-*trans* (min)			*t*_1/2_, reduction (min)
	DMSO	CKI buffer	Cellular assay medium	CKI buffer
4a	3.5	0.55	0.35	120
4b	1.7	0.28	0.18	54
4c	0.28	0.12	0.033	21
4d	18	0.65	0.82	33
4e	15	0.87	0.70	19
4f	43	1.2	0.93	25
4g	18	0.82	0.70	28
4h	68	ND^a^	ND^a^	5.9
4i	8.5	0.80	0.57	52
4j	10	0.70	0.58	55
4k	9.7	1.1	0.37	14
4l	36	ND^a^	0.73	9.1
4m	54	1.9	1.5	63
4n	180	ND^a^	36	6.7
4o	2.4	0.42	0.25	95
4p	4.1	0.77	0.45	79
4q	85	9.3	4.6	20

5a	78	3.1	2.0	13
5b	66^b^	ND^b^	ND^b^	92
5c	81	3.5	1.1	2.4
5d	46	1.8	1.0	83
5e	91	0.73	1.0	8.0
5f	710	18	7.3	29

a The half-life was not determined due to a constant absorption decrease upon dissolving the azopurine in CKI buffer or cellular assay medium.

b Compound 5b showed slow decomposition in DMSO.

Before evaluating photoswitchable azo-longdaysin derivatives in biological assays, the half-lives in the corresponding aqueous media were measured (CKI assay buffer and cellular assay medium, Table 1). In contrast to the results obtained in DMSO, and despite the incorporation of different substituents, a very fast thermal back-isomerization of all photoswitches was observed. The obtained half-lives varied from a few seconds (4c) to slightly more than half an hour (4n), and the observed trend was the same as in DMSO (Table 1). Di-*ortho*-fluoro substituents in 4q and 5f, di-*ortho*-chloro in 4n, and C(2)-amino substituents (5a–f) slowed down the thermal relaxation of the *cis*-isomer, but considering the length of the *in vitro* assay (3 h) and cellular assay (5 d), the back-isomerization was too fast for the photomodulation of the circadian period without constant or prolonged irradiation during the assay.

Despite the photophysical properties of azo-switches having been thoroughly investigated in the past,^58–60^ their chemical stability in biologically relevant media is still underexplored. However, to enable photocontrol in cells, tissues or living organisms, the chemical and metabolic stability of azobenzenes must be established. It is known that enzyme-^61,62^ or thiol-mediated^63^ azobenzene reduction can occur in cells, particularly by glutathione (GSH) that is present in cells at a concentration of up to 10 mM.^64,65^ In addition, a recent seminal work by Peifer, Herges and co-workers^66^ showed that photoswitchable CKIδ inhibitors based on imidazole- and thiazole-type heterocyclic azobenzenes undergo reduction to the corresponding hydrazines when exposed to thiols such as dithiothreitol (DTT) or GSH. Therefore, following the previous work and led by the finding that photoswitches 4h, 4l, 4n and 5b showed a continuous absorption decrease when dissolved in CKI assay buffer (Table 1), we tested all photoswitches for their reduction by DTT present in this buffer solution (Fig. 3A). Initially, the process was analyzed by ^1^H-NMR in DMSO-*d*_6_ solution of 4k, 4n and 5e (9 mM) and different quantities of DTT (0 eq., 1 eq., 2 eq., and 4 eq., Fig. 3B). The NMR spectrum of azopurine 4k did not change over time in the DTT-free sample. On the other hand, all three samples with DTT showed a second set of signals appearing after 10 min. In the presence of four equivalents of DTT, the starting azopurine 4k was almost completely (89%) converted to the corresponding hydrazine after 4 h. Additional analysis by UPLC-MS confirmed that the newly formed compound shows a mass increase of 2 units, consistent with the reduction product (Fig. S3†). Interestingly, when compound 4n was subjected to 1 eq. of DTT in DMSO-*d*_6_, bleaching due to azo bond reduction occurred instantaneously. This indicates that di-*ortho*-chloro substituents do not only play a stabilization role but also increase the susceptibility to reduction of the reported azobenzene system. This highlights the need for a systematic screening and better understanding of the chemical stability of heterocyclic azobenzenes under reductive conditions present in biological systems.^66^

**Fig. 3:**
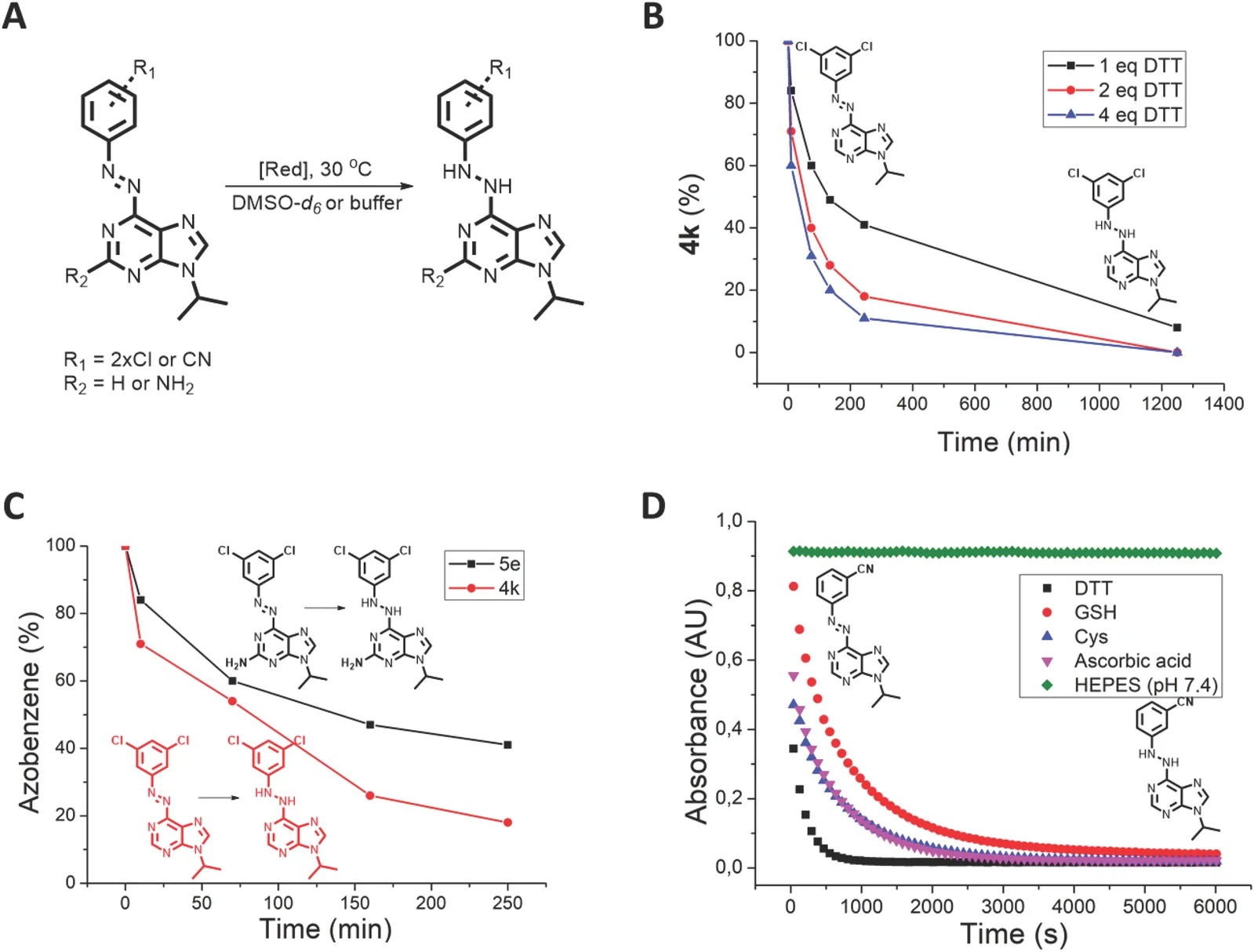
Reductive stability of azopurines. (A) The reduction process. (B) Reduction of 4k by different amounts of DTT followed by ^1^H-NMR in DMSO-*d*_6_ solution (25 °C, 9 mM 4k). (C) Comparison of the reduction rate of *trans*-4k and *trans*-5e by DTT (1 eq.). The reduction is followed by ^1^H-NMR in DMSO-*d*_6_ solution (25 °C, 9 mM). (D) Reduction of *trans*-4e followed by UV-Vis spectroscopy at 340 nm in buffer solution (100 mM HEPES, pH 7.4) without (green) or with different reducing agents (500 μM) – DTT (black), GSH (red), ascorbic acid (purple), and cysteine (blue).

Azopurines with a C(2)-amino group (5a–f) feature higher electron density on the aromatic rings. Therefore, we expected to see a slower reduction rate than for the corresponding C(2) unsubstituted azobenzenes. Testing the reduction of 4k and 5e with DTT confirmed our prediction (Fig. 3C). On the other hand, in an aqueous solution containing DTT (CKI buffer), both compounds are reduced so quickly that the difference is indistinguishable (Table 1).

Next, the reduction rate of all azobenzenes was measured in CKI buffer containing DTT (∼500 μM, Table 1). No clear correlation between the reduction rate and structure was observed. At the end of the measurement, all compounds were fully reduced, with reduction *t*_1/2_ ranging from ∼2 min (5c) to ∼2 h (4a). In that respect, measuring the thermal half-lives of the *cis*-isomers in the buffer was possible for almost all compounds except for 4h, 4l, 4n and 5b which underwent fast reduction competing with thermal back-isomerization. Since bleaching was also observed in cellular assay medium, we proceeded to the experimental testing of the stability of azopurine 4e in the presence of other reducing agents, such as GSH, cysteine and ascorbic acid and compared their reducing efficiency to DTT (Fig. 3D). Interestingly, the reduction was found to proceed with all reducing agents. The fastest reduction occurred in the case of DTT and the slowest with GSH, while ascorbic acid and cysteine showed similar, intermediate rates. The obtained results show the importance of testing every photoswitchable drug for the possible reduction with intracellular GSH, or reductive components present in *in vitro* buffer (such as DTT) that are used for preventing the oxidation of peptides and proteins.

### Photo-modulation of the CKI activity

Selected azopurines were tested *in vitro* in recombinant CKIα inhibition assays to assess their potency and the extent to which this potency can be photomodulated through azobenzene switching before reduction to the corresponding light-nonresponsive hydrazines occurs. Nine azopurines were selected for the screening of their activity in the dark (black bars, Fig. 4) and upon irradiation with green (*λ*_max_ = 530 nm) light (green bars, Fig. 4). All the compounds were initially tested at 20 μM concentration, assuming that after azologization the potency of longdaysin (IC_50_ value of 5.6 μM) was retained.^30^ Among the tested compounds (Fig. 4), azopurines 4b and 4c showed almost no inhibition of CKIα, similarly to di-*ortho*-methyl 4f and *meta*-carboxylic 4p azopurines. Irradiation with green light during the assay increased the activity of 4d, while 5e proved to be less potent after irradiation. The induced alteration in inhibition might be attributed to the difference in affinity between the two isomers. The relatively small difference in activity between the *cis*- and *trans*-isomer of 4d or 5e can be explained by the relatively fast reduction to the corresponding hydrazine by DTT present in the assay buffer, yielding the same light-nonresponsive product and preventing a significant difference in inhibition to be observed during the assay.

**Fig. 4:**
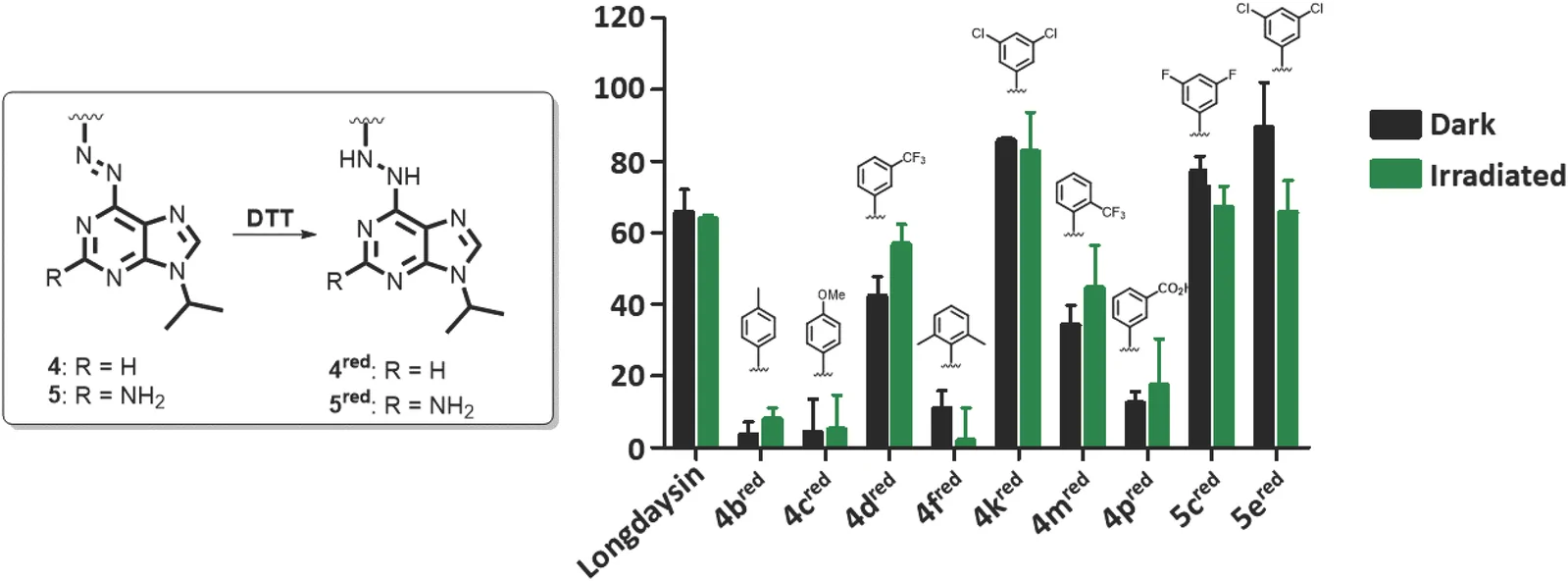
Degree of CKIα inhibition in the enzymatic assay starting with different azopurines (4 and 5, 20 μM, 30 °C) that get reduced to the corresponding hydrazines (4^red^ and 5^red^) under the assay conditions. Samples kept in the dark are shown in black, and samples irradiated during the assay in green. The results of the assays are mean ± SD of at least two independent measurements.

In order to test this hypothesis, the inhibition assay was performed starting with azobenzenes 4d and 5e, and their corresponding hydrazines (Fig. S5†). We observed that the inhibitory potency of azobenzenes and their reduced analogues was the same, confirming that reduction of the azo bond under the assay conditions occurs fast enough so that the difference in inhibition cannot be efficiently regulated with light. As a consequence, we attribute the biological effects observed in Fig. 4 and 5 to the reduced forms of photoswitches (4^red^ and 5^red^).

**Fig. 5:**
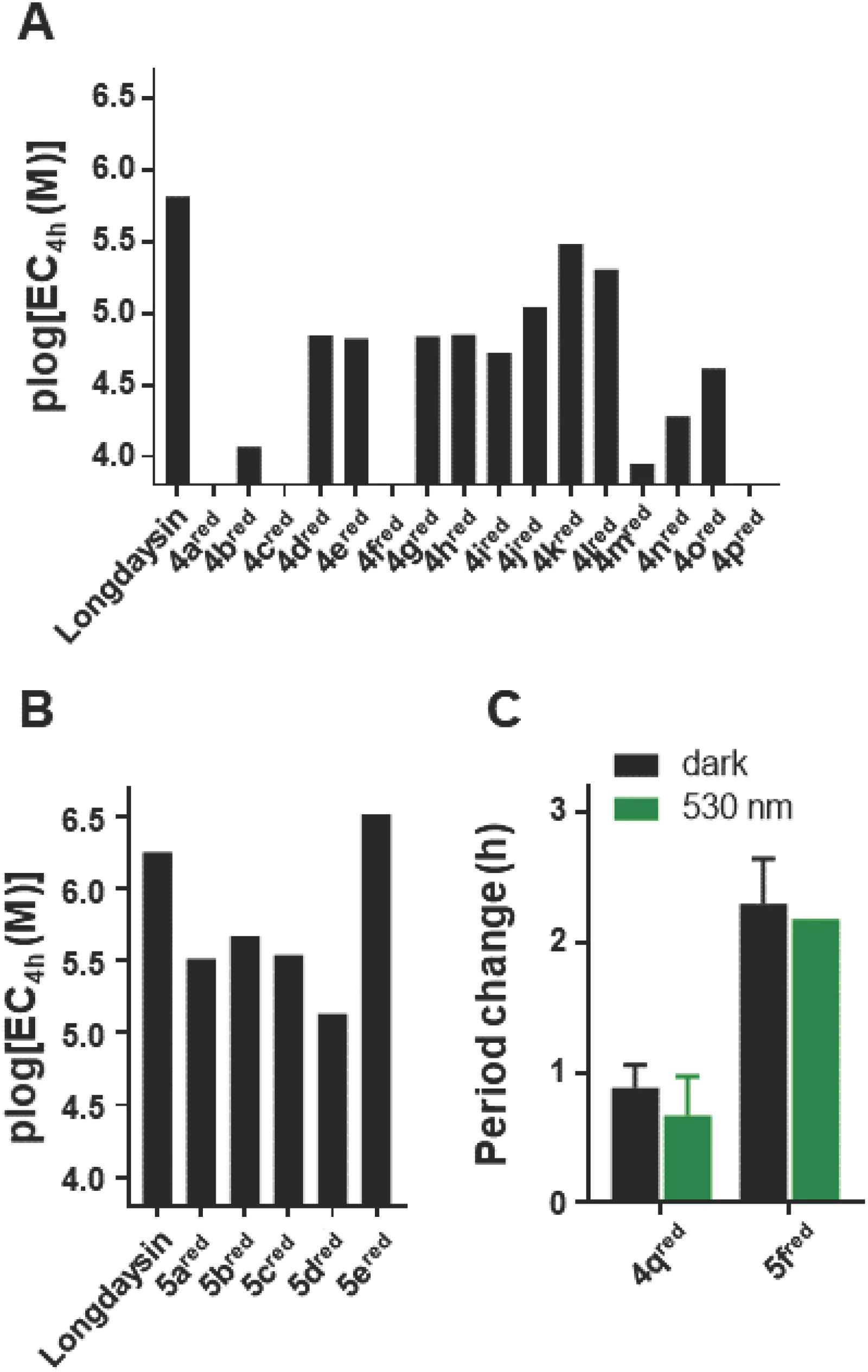
The effect of azopurines 4 and 5, which undergo reduction of the azo bond under the assay conditions to hydrazines 4^red^ and 5^red^, on the circadian period in human U2OS cells. Luminescence rhythms were monitored in the presence of various concentrations of compounds (12 points of 3-fold dilution series in DMSO; final 0.7% DMSO) in the dark (A and B) or upon irradiation with green light (*λ*_max_ = 530 nm) (C). In (A) and (B), [EC_4h_] (M) is the concentration required for a 4 h period change and it is plotted to compare the potency. [EC_4h_] values are calculated by fitting the 12-point dilution (*n* = 2) data with the exponential growth curve, and for 4a^red^, 4c^red^, 4f^red^ and 4p^red^ they could not be obtained due to significant toxicity of these compounds. In (C), the period change relative to DMSO (at 7.1 μM) is plotted.

### Modulation of the circadian period

Despite the observed reduction, we decided to test the azopurines for the modulation of the circadian period (Fig. 5). The assay employs human U2OS cells with a *Bmal1-dLuc* reporter (established at the University of California San Diego, USA) in DTT-free cellular medium.^37^ The experimental readout is based on chemiluminescence produced during the oxidative decarboxylation of luciferin. Luciferin is present in a high concentration (0.2 mM) in the cellular assay medium, and it strongly absorbs in the UV region. This prevents photoisomerization during the cellular assay with UV-light, which is generally used for the photoisomerization of azobenzene photoswitches.^5^ Gratifyingly, the possibility to isomerize the azopurines reported here with green light gives them an advantage for the application even in the presence of luciferin. Additionally, in contrast to UV-light, visible light is less cytotoxic, enabling longer irradiation prior to the performance of the cellular assay. Prolonged cellular irradiation (continuous or pulse) would maintain the presence of the *cis*-isomer for a longer time despite having a short half-life; however, again, it was not feasible in the current experimental setup (*vide infra*).

For the initial screening of the activity of the thermally stable *trans*-isomers, azopurines were dissolved in DMSO and applied to the cells in 3-fold dilution series. The chemiluminescence signal was measured every 100 min over 5 d and the concentrations required for 4 h circadian period lengthening from the DMSO control were plotted (Fig. 5). Compound 4a showed significantly reduced potency in comparison with longdaysin (Fig. 5A) and reduced reporter intensity (Fig. S6†), which likely arises from toxicity at higher concentrations. Similarly, azopurines substituted in the *para*-position (4b and 4c) showed a very small period change and caused reduction of the reporter intensity, indicating that introduction of a *para*-substituent is not beneficial for the biological activity and renders these compounds toxic. The *meta*-substituted azopurines exhibited strong period lengthening, with compound 5e having an even stronger effect than that of longdaysin (Fig. 5A and B). Incorporation of substituents at the *ortho*-position led to a decrease of period lengthening, but in contrast to the *para*-substituted azopurines, these compounds did not exhibit reduced reporter intensity (with the exception of 4f). Due to the beneficial effect of the *meta*-substituents, particular emphasis was put on introducing groups in this position. SAR analysis revealed that a polar carboxylic group in the *meta*-position fully suppressed the activity of 4p. The methyl group in 4o and the fluorine substituent in 4i slightly increased the activity, but the period change remained low. Moreover, 4o and 4p showed reduction of the reporter intensity. Introduction of CF_3_ or CN groups at the *meta*-position led to an almost equal increase of activity, while the chloro-substituent yielded the best circadian period modulator based on mono-*meta*-substituted azopurines. Interestingly, the cytotoxic effect was suppressed by introduction of these *meta*-substituents. The addition of the second *meta*-substituent contributed to a higher activity in comparison with the corresponding mono-*meta*-substituted compound.

In summary, comparing the circadian period lengthening effect of longdaysin and its direct azo-analogue 4d, it is evident that azologization of longdaysin decreased the potency, probably caused by the loss of an important interaction of the benzylic 6-NH group with CKIα.^67^ The hydrogen bond is possibly restored by reduction of the azo-group to the hydrazine 4d^red^. Thus, a reduced form of the 4d azo-analogue of longdaysin, at a concentration of 7.9 μM, exhibited only 2.2 h period lengthening in comparison with 11.5 h for longdaysin. However, the potency can be recovered by varying substituents on the benzene and purine moiety, and this strategy provided the reduced azopurine 5e^red^ with an activity even higher than that of longdaysin.

Finally, we investigated the possibility of light-induced circadian period modulation using selected compounds 4q and 5f. These two azopurines have a different C(2)-substituent, but the same benzene-ring substituents (di-*ortho*-fluoro). Both compounds were applied to cells in the dark (black bars, Fig. 5C) or irradiated with green light (*λ*_max_ = 530 nm) prior to dilution and then kept in the dark during the course of the assay (green bars, Fig. 5C). As expected, 5f^red^ showed a stronger effect on the circadian period modulation than that of 4q^red^. Nevertheless, a clear light-induced effect was not observed. This can be explained by both a short *cis*-isomer half-life in the cellular assay medium and a fast reduction to the corresponding hydrazines 4q^red^ and 5f^red^ that are not responsive to light.

As a result of the assay's length (5 d) and a possible fast reduction by intracellular GSH, we assume that the observed period lengthening effect mostly arises from the corresponding hydrazine. Due to, *inter alia*, unavoidable endogenous GSH production by the cells, it was not possible to prove if the circadian period can be additionally modulated by light. Nevertheless, a comprehensive library of compounds revealed a molecule with stronger period lengthening than that of longdaysin.

## Conclusion

A library of azopurines was synthesized and photochemically characterized and their chemical stability in the biological environment was investigated. Furthermore, their biologically activity as CKIα kinase inhibitors was tested in enzyme activity and circadian period modulation assays.

The two-step one-pot synthesis provided a quick and efficient access to the library of differently substituted azopurines. Photochemical characterization revealed that green light can be utilized for photo-isomerization, yielding moderate PSS distributions. The thermal stability of the *cis*-isomer was measured in DMSO and aqueous media, showing that the rate of the *cis*-to-*trans* isomerization can be efficiently manipulated by varying substituents on the benzene ring or at the C(2)-position. While a broad range of half-lives was obtained in DMSO, thermal isomerization in aqueous media occurred on shorter timescales, rendering these photoswitches unsuitable for light modulation in long-term circadian rhythm experiments. Furthermore, azopurines were tested for their sensitivity to reduction with reducing agents commonly present in cells (GSH) or used for stabilization of proteins (*e.g.*, DTT). Both in DMSO and aqueous media, the reduction took place, and the rate was similar for all the compounds. The reduction process was clean, yielding pure light-nonresponsive hydrazines. Despite the observed reduction, all the compounds were tested for CKIα inhibition and circadian period modulation. Photo-modulation of the CKIα inhibition activity was observed, but due to the rapid DTT-mediated reduction of the azo functional group, the effect was not significantly pronounced and probably can be attributed to a different reduction rate of the photo-isomers. Interestingly, the cellular assay screening yielded compound 5e^red^ with stronger period lengthening than that of longdaysin. At a concentration of 7.9 μM, azopurine 5e^red^ displayed a 12 h period-lengthening effect, in comparison with 9.2 h of longdaysin. However, the reduction rate and the nature of the circadian cellular assay prevented photo-modulation of the circadian period and CKIα inhibition.

In summary, a comprehensive SAR analysis of the ability of reduced azopurines to modulate the circadian period was performed. The reduction study and screening of reducing agents revealed DTT as the most potent reducing agent for the azo group. These results support previous findings by Peifer, Herges and co-workers,^66^ confirming that DTT is a crucial reagent to be used in photopharmacology to test the biological stability of heteroazoarenes towards reduction. This work reveals challenges associated with azobenzene-type photomodulators of circadian rhythms in the broader context of photopharmacology. It also emphasizes the importance of achieving a long half-life, low fatigue, high photostationary state distributions (PSD) and a chemically stable photoswitch when long-term biological light modulation is required.

## Conflicts of interest

There are no conflicts to declare.

## Supplementary Material

OB-019-D1OB00014D-s001
