# Supplementary material for: Reductive stability evaluation of 6-azopurine photoswitches for the regulation of CKIα activity and circadian rhythms
Source: Org Biomol Chem. 2021 Feb 19;19(10):2312–21. doi: 10.1039/d1ob00014d (PMC13725694; doi:10.1039/d1ob00014d)
Supplement: Supplementary file 1 [file OB-019-D1OB00014D-s001.pdf]

Electronic Supplementary Material (ESI) for Organic & Biomolecular Chemistry.
This journal is © The Royal Society of Chemistry 2021

Supporting Information for:

Reductive stability evaluation of 6-azopurine photoswitches for the regulation of CKIα activity and circadian rhythm

Dušan Kolarski,[a] Akiko Sugiyama,[b] Theo Rodat,[c] Albert Schulte,[a] Christian Peifer,[c] Kenichiro Itami,[b] Tsuyoshi Hirota,*[b] Ben L. Feringa,*[a] and Wiktor Szymanski*[a,d]

[a] Stratingh Institute for Chemistry, University of Groningen, Nijenborgh 4, 9747 AG, Groningen (The Netherlands)

[b] Institute of Transformative Bio-Molecules (WPI-ITbM), Nagoya University, Furo-cho, Chikusa, Nagoya 464-8601 (Japan)

[c] Department of Pharmaceutical and Medicinal Chemistry, Christian-Albrechts-University of Kiel, Gutenbergstraße 76, 24118 Kiel (Germany)

[d] Medical Imaging Center, University Medical Center Groningen, University of Groningen, Hanzeplein 1, 9713 GZ Groningen (The Netherlands)

Contents

EXPERIMENTAL DETAILS 3

REDUCTION OF AZOBENZANES FOLLOWED BY UV-VIS SPECTROSCOPY 6

REDUCTION OF AZOBENZANES FOLLOWED BY HPLC-MS 7

EXPERIMENTAL SECTION 8

PHOTOCHEMISTRY OF AZOPURINES 11

KINASE ASSAY RESULTS 12

REPORTED INTENSITY 12

NMR DATA 13

REFERENCES 23

EXPERIMENTAL DETAILS

General reagent information

The reaction progress was monitored by TLC. Starting materials, reagents and solvents were purchased from Sigma–Aldrich, Acros, Fluka, Fischer, TCI or Combi-Blocks and were used as received, unless stated otherwise. Solvents for the reactions were of quality puriss., p.a. For aqueous solutions, deionized water was used.

General considerations

Thin Layer Chromatography analyses were performed on commercial Kieselgel 60, F254 silica gel plates with fluorescence-indicator UV254 (Merck, TLC silica gel 60 F254). For detection of components, UV light at λ = 254 nm or λ = 365 nm was used. Alternatively, oxidative staining using aqueous basic potassium permanganate solution ($KMnO_4$) or aqueous acidic cerium phosphomolybdic acid solution (Seebach's stain) was used. Drying of solutions was performed with $MgSO_4$ and volatiles were removed with a rotary evaporator (Büchi, R-300).

General analytical information

Nuclear Magnetic Resonance spectra were measured with an Agilent Technologies 400-MR (400/54 Premium Shielded) spectrometer (400 MHz). All spectra were measured at room temperature (22–24 °C). Chemical shifts for the specific NMR spectra were reported relative to the residual solvent peak [in ppm; $CDCl_3$: δ_H = 7.26; $CDCl_3$: δ_C = 77.16; d_6-DMSO: δ_H = 2.50; d_6-DMSO: δ_C = 39.52]. The multiplicities of the signals are denoted by s (singlet), d (doublet), t (triplet), q (quartet), hept (heptet), m (multiplet), br (broad signal). All ^{13}C-NMR spectra are ^{1}H-broadband decoupled.

High-resolution mass spectrometric measurements were performed using a Thermo scientific LTQ OrbitrapXL spectrometer with ESI ionization.

Melting points were recorded using a Stuart analogue capillary melting point SMP11 apparatus.

All the reactions were performed in CEM Discover SP-D microwave reactor. Room temperature UV-Vis absorption spectra were recorded on an Agilent 8453 UV-Visible Spectrophotometer using Uvasol grade solvents. UPLC-MS measurements were done using ThermoFischer Scientific Vanquish UPLC System on C18 column.

Irradiation experiments were performed with LED system (λ_{max} = 530 nm, 3x Nichia NCSG219B-V1, 3x550 mW, Sahlmann Photochemical Solutions).

Cell Line

Bmal1-dLuc U2OS cells were established at University of California San Diego (USA) and maintained as described previously.[1]

NMR experiments

Nuclear Magnetic Resonance (NMR) spectra were measured with an Agilent Technologies 400-MR (400/54 Premium Shielded) spectrometer (400 MHz). All spectra were measured at room temperature (22-24 °C).

Photoisomerization studies were conducted in the NMR tubes in DMSO-d_6 solutions containing 1 mg/mL of compounds. Irradiation with green light (λ_{max} = 530 nm, 3x Nichia NCSG219B-V1, 3x550 mW, Sahlmann Photochemical Solutions) was applied until the PSD was reached.

The reduction rate was followed in DMSO-d_6 solutions at the concentration of 9 mM. Solutions of the azo-compound and DTT were prepared in the dark and stirred at room temparature. After indicated time, aliquotes were taken and the NMR spectrum of the sample was recorded.

UV-Vis measurements

UV-Vis absorption spectra were recorded on an Agilent 8453 UV-Visible Spectrophotometer using Uvasol grade solvents.
Thermal *cis*-to-*trans* isomerization was followed in three different media: DMSO (40 µM, 25 °C), CKI enzyme assay buffer (40 µM, 30 °C) containing ~100 µM dithiothreitol (DTT) and cellular assay medium (40 µM, 35 °C). The DMSO solution (2 mM) was irradiated with green light until it reached the PSD, and then diluted with the corresponding medium. Recovery of absorbance at λ_{max} was followed over time and the half-life was obtained using exponential fit in Origin.
The reduction rate of azobenzenes (40 µM) in CKI buffer containing 500 µM DTT (30 °C) was followed by measuring absorbance at λ_{max} over the time. Half-life of reduction was obtained by exponential fit in Origin.

Evaluation of the circadian period modulation

Effects of compounds on cellular circadian rhythms were analyzed as described previously[1] with modifications. Stable U2OS reporter cells harboring *Bmal1-dLuc* reporter were suspended in phenol red-free culture medium [DMEM (D2902, Sigma) supplemented with 10% fetal bovine serum, 3.5 mg/mL D-glucose, 3.7 mg/mL sodium bicarbonate, 0.29 mg/mL L-glutamine, 100 units/mL penicillin, and 100 µg/mL streptomycin] and plated onto a white, solid-bottom 384-well plates at 30 µL (3,000 cells) per well. After 2 days, 40 µL of phenol red-free explant medium [DMEM (D2902, Sigma) supplemented with 2% B27 (Gibco), 10 mM HEPES, 3.5 mg/mL D-glucose, 0.38 mg/mL sodium bicarbonate, 0.29 mg/mL L-glutamine, 100 units/mL penicillin, 100 µg/mL streptomycin, and 0.2 mM luciferin; pH 7.2] was dispensed into each well, followed by the application of 0.5 µL of compounds (dissolved in DMSO; final 0.7% DMSO; pre-irradiated with green light for 60 min). Luminescence was recorded every 100 min in a microplate reader, Infinite M200Pro (Tecan) or Synergy2 (BioTek).
N.B. The cellular circadian assay requires a continuous measurement of the weak luminescent signal. Therefore, it is crucial to keep the plate in dark inside of the plate reader. This precludes any continuous or pulsed irradiation during the assay.

Circadian parameters calculation

Raw luminescence data are detrended with a first-order polynomial curve and then fitted to a damping sinusoid curve:

$$Y(t) = A*\sin(2\pi ft + \phi)*e^{-t/\tau} + C$$

A = amplitude; f = frequency (1/period); φ, phase; τ, damping constant; C, offset.

Enzymatic assay with CKIα

The assays were performed on a white, solid-bottom 384-well plates. The total volume for the reaction is 10.5 µl. Firstly, a solution of CKI and peptide was added to the bottom of the well (9 µL). Next, a corresponding solution of the compound (0.5 µL, final 5% DMSO) followed by 50 µM ATP solution

(1 μL) were pipetted into the upper corners of each well. The enzymatic reaction was started by spinning down the plate (3000 rpm, 2 min).

By employing this method, all reactions were started at the same time, minimizing variance between different samples. Incubation for 3 h at 30 °C allowed for the enzymatic phosphorylation of the substrate peptide. As the reaction started, half of the wells were irradiated with green light and the other half was kept in dark. After the incubation, 10 μL Kinase Glo® (Promega) was applied into the wells and the luminescent signal was recorded by a plate reader (BioTek Synergy H1 or Cytation).

In the experiment where the activity of azobenzenes and their corresponding hydrazines was compared, the DMSO solution of the azobenzene was treated with DTT (2 equiv) until the colour disappeared and a colourless solution persisted (~30 min). The DMSO and longdaysin controls were also treated the same way as azobenzenes, and separately measured from DTT-free stock solutions.

REDUCTION OF AZOBENZANES FOLLOWED BY UV-VIS SPECTROSCOPY

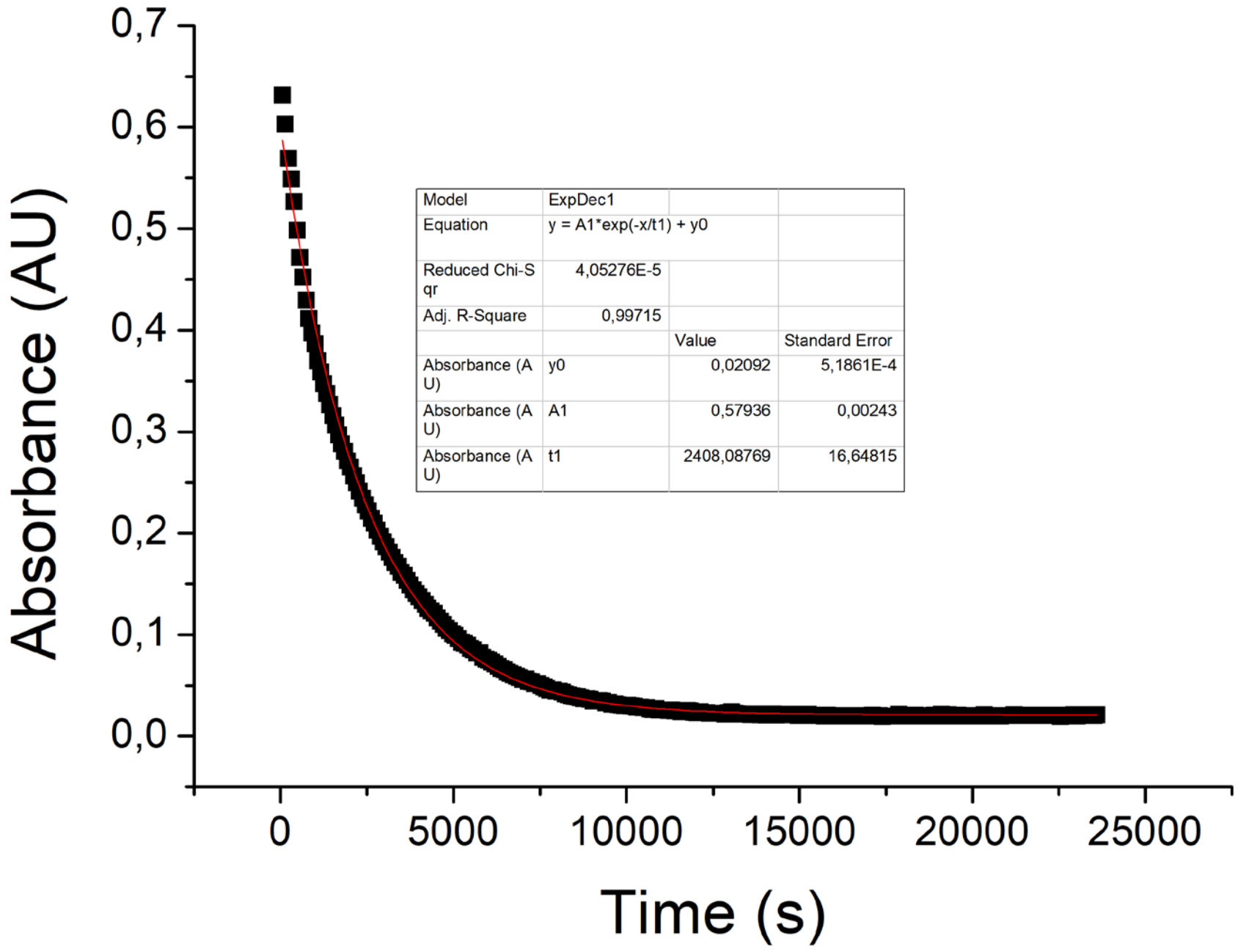

Fig. S1. Representative example of azoadenine (**4g**, 40 µM) reduction by DTT in kinase assay buffer (500 µM, 30 °C).

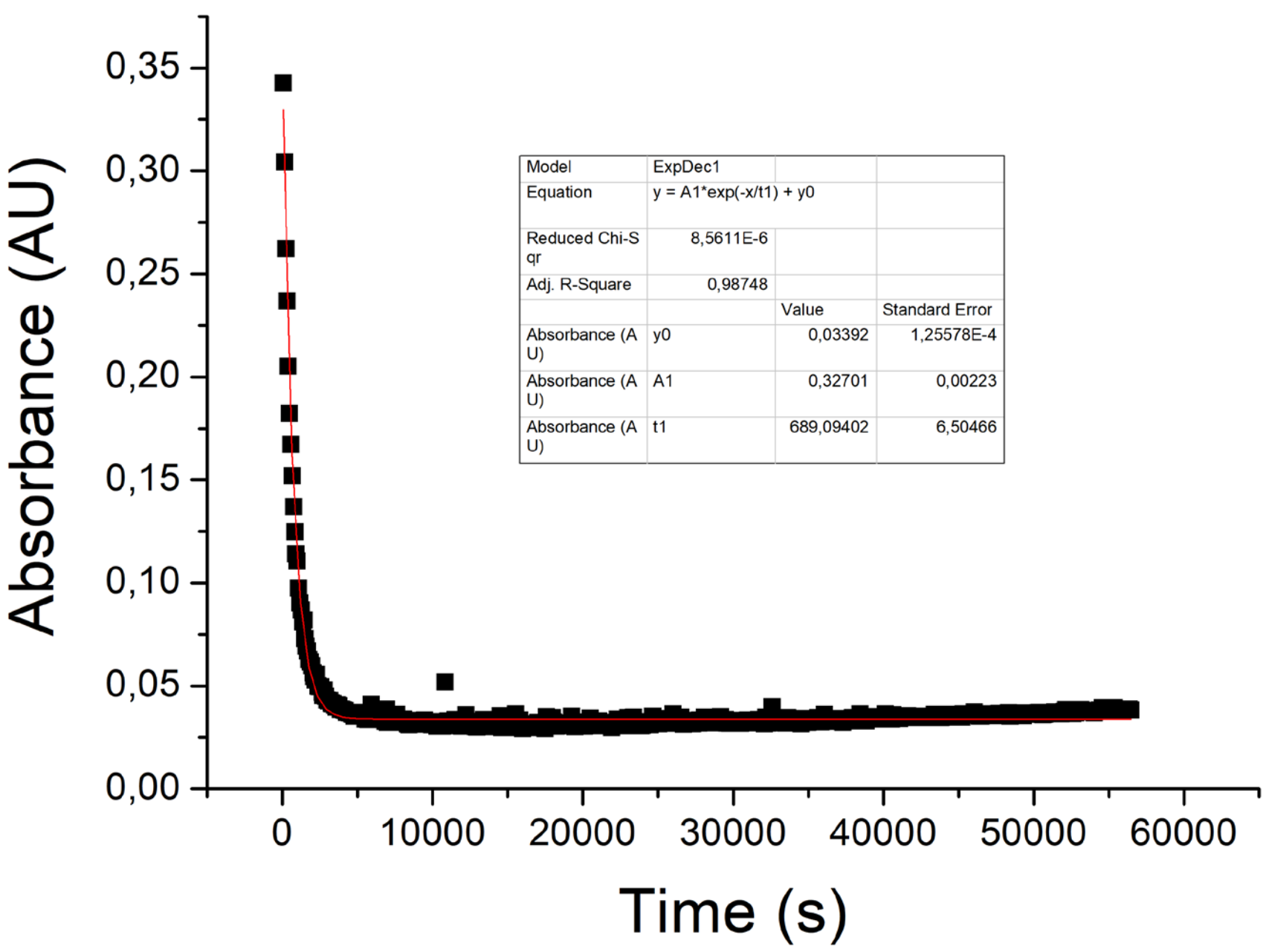

Fig. S2. Representative example of azoguanine (**5e**, 40 µM) reduction by DTT in kinase assay buffer (500 µM, 30 °C).

REDUCTION OF AZOBENZANES FOLLOWED BY HPLC-MS

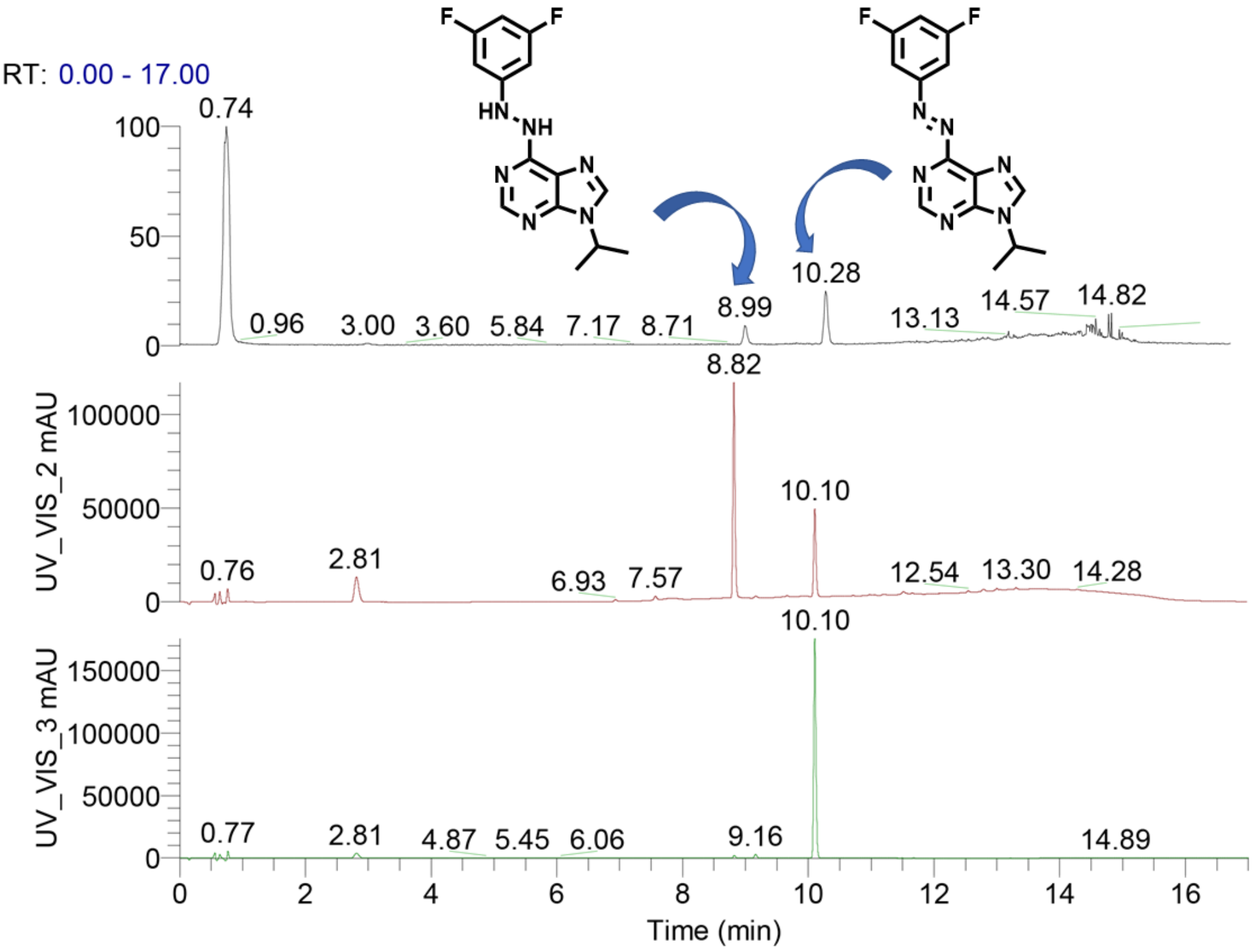

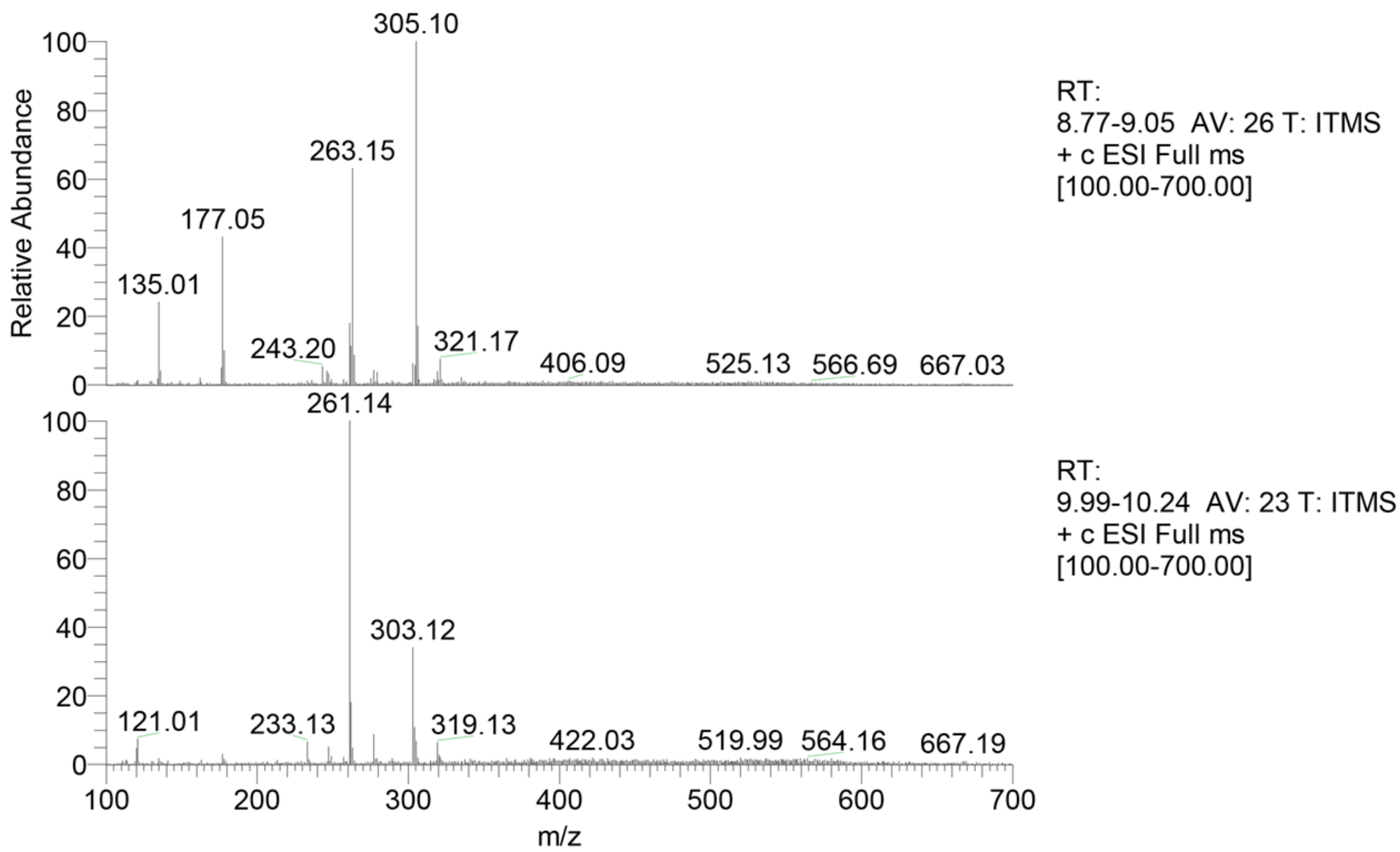

Fig. S3. LCMS traces of **4g** sample in the kinase assay buffer (40 µM) showing the presence of hydrazine (RT = 8.99 min, ms = 305.10) and azobenzene (RT = 10.28 min, ms = 303.12). Also, traces of both compounds can be seen under 254 nm (UV_VIS_2) while at 330 nm only the azobenzene form can be detected (UV_VIS_3).

EXPERIMENTAL SECTION

General Procedure for the Synthesis of 6-Azoapurines 4a-p. [2]

The reaction was carried out using a microwave vessel (10 mL) equipped with a magnetic stirring bar, in the presence of air and using CEM Discover SP-D microwave reactor. 6-Chloro-9-isopropyl-9H-purine **2** (59 mg, 0.30 mmol, 1.0 equiv), hydrazine (0.36 mmol, 1.2 equiv), DIPEA (0.26 mL, 1.5 mmol, 5 equiv in case of hydrazine, or 0.31 mL, 1.8 mmol, 6 equiv in case of hydrazine hydrochloride) and *n*-BuOH (2.0 mL) were added in sequence. The resulting mixture was reacted under microwave irradiation (200 W) at 150 °C for 1-2 h. After the substitution was completed (followed by TLC), the reaction mixture was exposed to pure oxygen for 30 min – 24 h. After the oxidation reaction was finished (followed by TLC), the solvent was removed under reduced pressure and the product was purified by flash column chromatography (SiO_2, DCM/MeOH 98:2) to give **4a-r** as the orange-red solids.
Additional recrystallization was performed from ethyl acetate/pentane in case of **4p**.
Complete characterization data for compounds **4a**, **4b**, **4c**, **4e**, **4f**, **4g**, **4h**, **4i**, **4j**, **4k**, **4m**, **4n**, **4o**, and **4p** can be found in reference 34. [2]

(*E*)-9-isopropyl-6-((3-(trifluoromethyl)phenyl)diazenyl)-9H-purine (**4d**)

Dark red solid; Yield: 82 mg (0.3 mmol, 99%); m.p. = 111-113 °C; ^{1}H NMR (400 MHz, $CDCl_3$) δ 9.09 (s, 1H), 8.47 (s, 1H), 8.39 (d, J = 8.7 Hz, 1H), 8.33 (s, 1H), 7.85 (d, J = 8.6 Hz, 1H), 7.72 (t, J = 8.2 Hz, 1H), 5.05 (hept, J = 7.0 Hz, 1H), 1.72 (dd, J = 6.8, 0.8 Hz, 6H) ppm; ^{13}C NMR (101 MHz, $CDCl_3$) δ 156.82, 155.11, 152.84, 152.24, 144.92, 131.95 (q, J = 33.3 Hz), 129.88, 129.39 (q, J = 3.7 Hz), 127.34, 127.15, 123.61 (q, J = 272.6 Hz), 120.94 (q, J = 3.9 Hz), 48.05, 22.52 ppm; ^{19}F NMR (376 MHz, $CDCl_3$) δ -62.77 (s) ppm; IR (ATR) $\tilde{\nu}$ 3060, 2984, 1867, 1739, 1373, 1328, 1222, 1124, 1062, 909, 810 cm^{-1}; HRMS (ESI^{+}) calc. for $C_{15}H_{14}N_6F_3$ [M+H]$^{+}$: 335.1227, found: 335.1228.

(*E*)-6-((3,5-bis(trifluoromethyl)phenyl)diazenyl)-9-isopropyl-9H-purine (**4l**)

Dark red solid; Yield: 123 mg (0.3 mmol, 93%); m.p. = 112-114 °C; ^{1}H NMR (400 MHz, $CDCl_3$) δ 9.11 (s, 1H), 8.65 (s, 2H), 8.38 (s, 1H), 8.09 (s, 1H), 5.06 (hept, J = 6.8 Hz, 1H), 1.73 (d, J = 6.8 Hz, 6H) ppm; ^{13}C NMR (101 MHz, $CDCl_3$) δ 156.30, 155.29, 152.93, 152.23, 145.39, 133.00 (q, J = 34.3 Hz), 125.98 – 125.78 (m), 124.09 (q, J = 3.7 Hz), 121.47, 48.26, 22.49 ppm; ^{19}F NMR (376 MHz, $CDCl_3$) δ -62.94 ppm; IR (ATR) $\tilde{\nu}$ 3105, 2981, 1817, 1586, 1499, 1454, 1364, 1274, 1169, 1125, 1013, 946, 907 cm^{-1}; HRMS (ESI^{+}) calc. for $C_{16}H_{13}N(6)F_6$ [M+H]$^{+}$: 403.1100, found: 403.1094.

(*E*)-9-isopropyl-6-((2-(trifluoromethyl)phenyl)diazenyl)-9H-purine (**4m**)

Dark red solid; Yield: 99 mg (0.3 mmol, 89%); m.p. = 104-106 °C; ^{1}H NMR (400 MHz, $CDCl_3$) δ 9.09 (s, 1H), 8.34 (s, 1H), 7.98 – 7.83 (m, 2H), 7.75 – 7.62 (m, 2H), 5.04 (hept, J = 6.8 Hz, 1H), 1.71 (d, J = 6.8 Hz, 6H) ppm; ^{13}C NMR (101 MHz, $CDCl_3$) δ 157.72, 154.90, 152.14, 150.09, 144.76, 132.57, 132.24, 129.53 (q, J = 31.7 Hz), 126.66 (q, J = 5.4 Hz), 126.55, 123.70 (q, J = 274.2 Hz), 116.62, 47.91, 22.52 ppm; ^{19}F NMR (376 MHz, $CDCl_3$) δ -57.32 ppm; IR (ATR) $\tilde{\nu}$ 3351, 3044, 2979, 2940, 1596, 1571, 1492, 1394, 1314, 1215, 1144, 1120, 1052, 882, 772 cm^{-1}; HRMS (ESI^{+}) calc. for $C_{15}H_{14}N_6F_3$ [M+H]$^{+}$: 335.1227, found: 335.1228.

(*E*)-6-((2,6-difluorophenyl)diazenyl)-9-isopropyl-9H-purine (**4q**)

Dark red solid; Yield: 73 mg (0.24 mmol, 80%); m.p. = 112-114 °C; ^{1}H NMR (400 MHz, $CDCl_3$) δ 9.08 (s, 1H), 8.31 (s, 1H), 7.46 (tt, *J* = 8.4, 5.8 Hz, 1H), 7.15 – 7.05 (m, 2H), 5.03 (hept, *J* = 6.8 Hz, 1H), 1.70 (d, *J* = 6.8 Hz, 6H) ppm; ^{13}C NMR (101 MHz, $CDCl_3$) δ 157.52 (d, *J* = 3.7 Hz), 157.34, 155.09, 154.89 (d, *J* = 3.7 Hz), 152.17, 145.06, 133.12 (t, *J* = 10.6 Hz), 126.59, 112.73 (dd, *J* = 20.3, 3.8 Hz), 47.94, 22.53 ppm; ^{19}F NMR (376 MHz, $CDCl_3$) δ -118.31 (dd, *J* = 9.5, 5.9 Hz) ppm; IR (ATR) $\tilde{\nu}$ 2980, 1565, 1487, 1467, 1389, 1324, 1219, 1029, 984, 789 cm^{-1}; HRMS (ESI^{+}) calc. for $C_{14}H_{13}N_6F_2$ [M+H]$^{+}$: 303.1164, found: 303.1166.

General Procedure for the Synthesis of 6-Azoapurines 5a-f. [2]

The reaction was carried out using a microwave vessel (10 mL) equipped with a magnetic stirring bar, in the presence of air and using CEM Discover SP-D microwave reactor. 6-Chloro-9-isopropyl-*9H*-purin-2-amine **3** (65 mg, 0.30 mmol, 1.0 equiv), hydrazine (0.36 mmol, 1.2 equiv), DIPEA (0.26 mL, 1.5 mmol, 5 equiv in case of hydrazine, or 0.31 mL, 1.8 mmol, 6 equiv in case of hydrazine hydrochloride) and *n*-BuOH (2.0 mL) were added in sequence. The resulting mixture was reacted under microwave irradiation (200 W) at 180 °C for 1-3 h. After the substitution was completed (followed by TLC), the reaction mixture was exposed to a pure oxygen for 30 min – 18 h. After the reaction was finished (followed by TLC), the solvent was removed under reduced pressure and the product was purified by flash column chromatography (SiO_2, DCM/MeOH 96:4) to give **5a-f** as the red-brown solids.

When needed, additional recrystallization was done from ethyl acetate/pentane.

A complete characterization data for compounds **5c**, **5d**, and **5e** can be found in reference 34.[2]

(*E*)-9-isopropyl-6-((3-(trifluoromethyl)phenyl)diazenyl)-9H-purin-2-amine (**5a**)

Dark red solid; Yield: 99 mg (0.28 mmol, 92%); m.p. = 117-119 °C; ^{1}H NMR (400 MHz, $CDCl_3$) *δ* 8.41 (s, 1H), 8.33 (d, *J* = 8.0 Hz, 1H), 8.00 (s, 1H), 7.81 (d, *J* = 7.8 Hz, 1H), 7.68 (t, *J* = 7.9 Hz, 1H), 5.32 (s, 2H), 4.80 (hept, *J* = 6.8 Hz, 1H), 1.61 (d, *J* = 6.8 Hz, 6H) ppm; ^{13}C NMR (101 MHz, $CDCl_3$) δ 159.31, 157.62, 156.89, 152.79, 142.35, 131.82 (q, *J* = 33.3 Hz), 129.76, 129.12 (q, *J* = 3.6 Hz), 127.16, 123.62 (q, *J* = 272.7 Hz), 121.27, 120.83 (q, *J* = 3.9 Hz), 47.05, 22.41 ppm; ^{19}F NMR (376 MHz, $CDCl_3$) δ -62.76 ppm; IR (ATR) $\tilde{\nu}$ 3282, 3175, 3094, 2975, 1710, 1620, 1576, 1453, 1394, 1328, 1217, 1170, 1119, 1061, 993, 906, 804 cm^{-1}; HRMS (ESI^{+}) calc. for $C_{15}H_{15}N_7F_3$ [M+H]$^{+}$: 350.1336, found: 350.1337.

(*E*)-6-((3,5-bis(trifluoromethyl)phenyl)diazenyl)-9-isopropyl-9H-purin-2-amine (**5b**)

Dark red solid; Yield: 73 mg (0.18 mmol, 57%); m.p. = 199-202 °C; ^{1}H NMR (400 MHz, $CDCl_3$) *δ* 9.72 (s, 1H), 8.59 (s, 2H), 8.07 (s, 1H), 5.24 (s, 2H), 4.74 (h, *J* = 7.0 Hz, 1H), 1.60 (d, *J* = 6.9 Hz, 6H) ppm; ^{13}C NMR (101 MHz, $CDCl_3$) δ 159.31, 157.62, 156.89, 152.79, 142.35, 131.82 (q, *J* = 33.3 Hz), 129.76, 129.12 (q, *J* = 3.6 Hz), 127.16, 123.62 (q, *J* = 272.7 Hz), 121.27, 120.83 (q, *J* = 3.9 Hz), 47.05, 22.41 ppm; ^{19}F NMR (376 MHz, $CDCl_3$) δ -62.76 ppm; IR (ATR) $\tilde{\nu}$ 3500, 3278, 3162, 1714, 1628, 1615, 1501, 1435, 1367, 1277, 1199, 1129, 904 cm^{-1}; HRMS (ESI^{+}) calc. for $C_{16}H_{14}N_7F_6$ [M+H]$^{+}$: 418.1209, found: 418.1207.

(*E*)-6-((2,6-difluorophenyl)diazenyl)-9-isopropyl-9H-purin-2-amine (**5f**)

Dark red solid; Yield: 70 mg (0.22 mmol, 72%); m.p. = 206-208 °C; ^{1}H NMR (400 MHz, $CDCl_3$) δ 7.99 (s, 1H), 7.43 (tt, *J* = 8.5, 5.8 Hz, 1H), 7.13 – 7.03 (m, 2H), 5.30 (s, 2H), 4.80 (hept, *J* = 6.8 Hz, 1H), 1.61 (d, *J* = 6.8 Hz, 6H) ppm; ^{13}C NMR (101 MHz, $CDCl_3$) δ 159.22, 157.87, 157.43 (d, *J* = 3.7 Hz), 156.94, 154.81 (d, *J* = 3.9 Hz), 142.52, 132.78 (t, *J* = 10.5 Hz), 120.95, 112.66 (dd, *J* = 20.4, 3.7 Hz), 46.99, 22.43 ppm; ^{19}F NMR (376 MHz, $CDCl_3$) δ -118.49 (dd, *J* = 9.9, 6.0 Hz) ppm; IR (ATR) $\tilde{\nu}$ 3510, 3451, 3322, 3206, 2975, 1612, 1573, 1461, 1274, 1223, 1037, 1000, 794 cm^{-1}; HRMS (ESI^{+}) calc. for $C_{14}H_{14}N_7F_2$ $[M+H]^{+}$: 318.1273, found: 318.1275.

PHOTOCHEMISTRY OF AZOPURINES

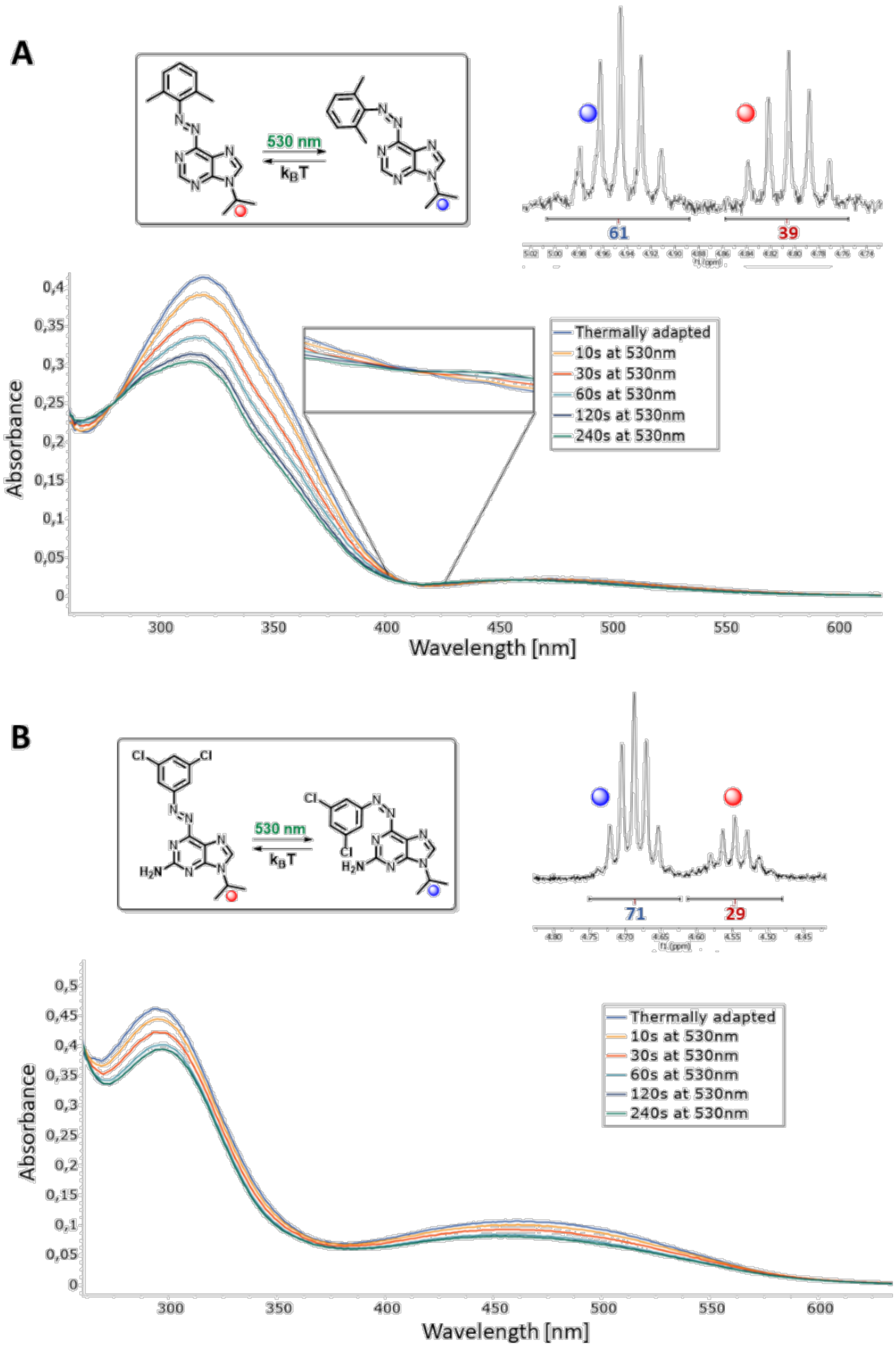

Fig. S4. Photochemical evaluation of **(A)** compound **4f**, and **(B)** compound **5e.** ^{1}H-NMR spectroscopy was used to determine the PSDs of both compounds (DMSO-d_6, 1 mg/mL, 25 °C), which were reached after irradiation with 530 nm light for 2 h. PSS (**4f**) = 61% *cis*, and PSS (**5e**) = 71% *cis*. UV-Vis studies of the photochemical isomerization were followed in a ~20 µM DMSO solution at 25 °C. Photostationary state was reached after 4 min upon irradiation with λ = 530 nm light (measured points: thermally adapted – 0 s, 10 s, 30 s, 60 s, 120 s, and 240 s).

KINASE ASSAY RESULTS

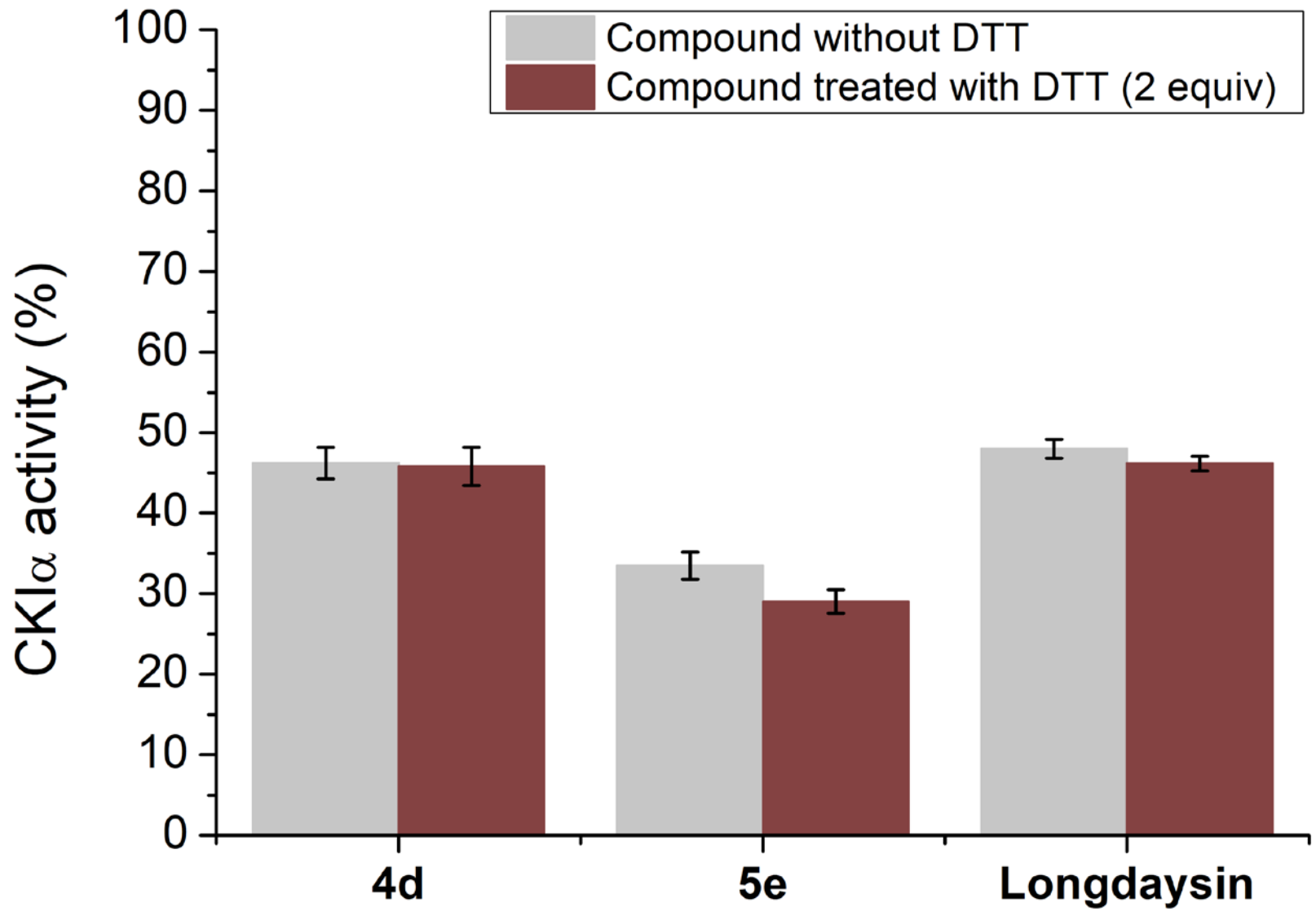

Fig. S5. The activity of CKIα in the enzymatic assay containing **4d** and **5e** azopurines (20 µM, 30 °C, grey) or corresponding hydrazines (20 µM, 30 °C, dark red) obtained by reduction with DTT (40 µM). The longdaysin control with (grey) and without (dark red) DTT is shown (20 µM, 30 °C). The results of the assays are mean ± SD of at least two independent measurements.

REPORTED INTENSITY

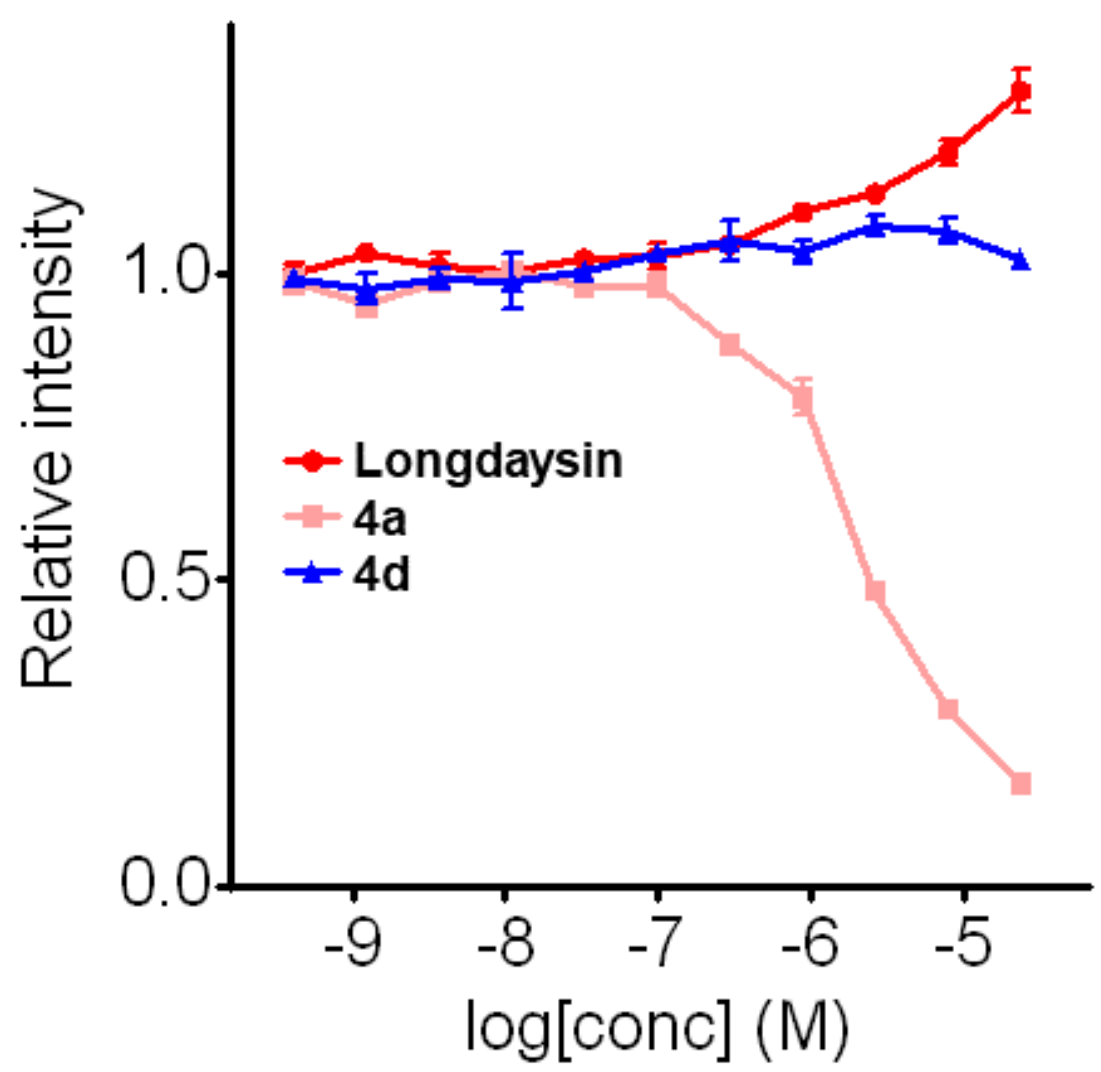

Fig. S6. Effect of longdaysin and azopurines (**4a** and **4d**) on the relative luminescent intensity in human U2OS cells.

NMR DATA

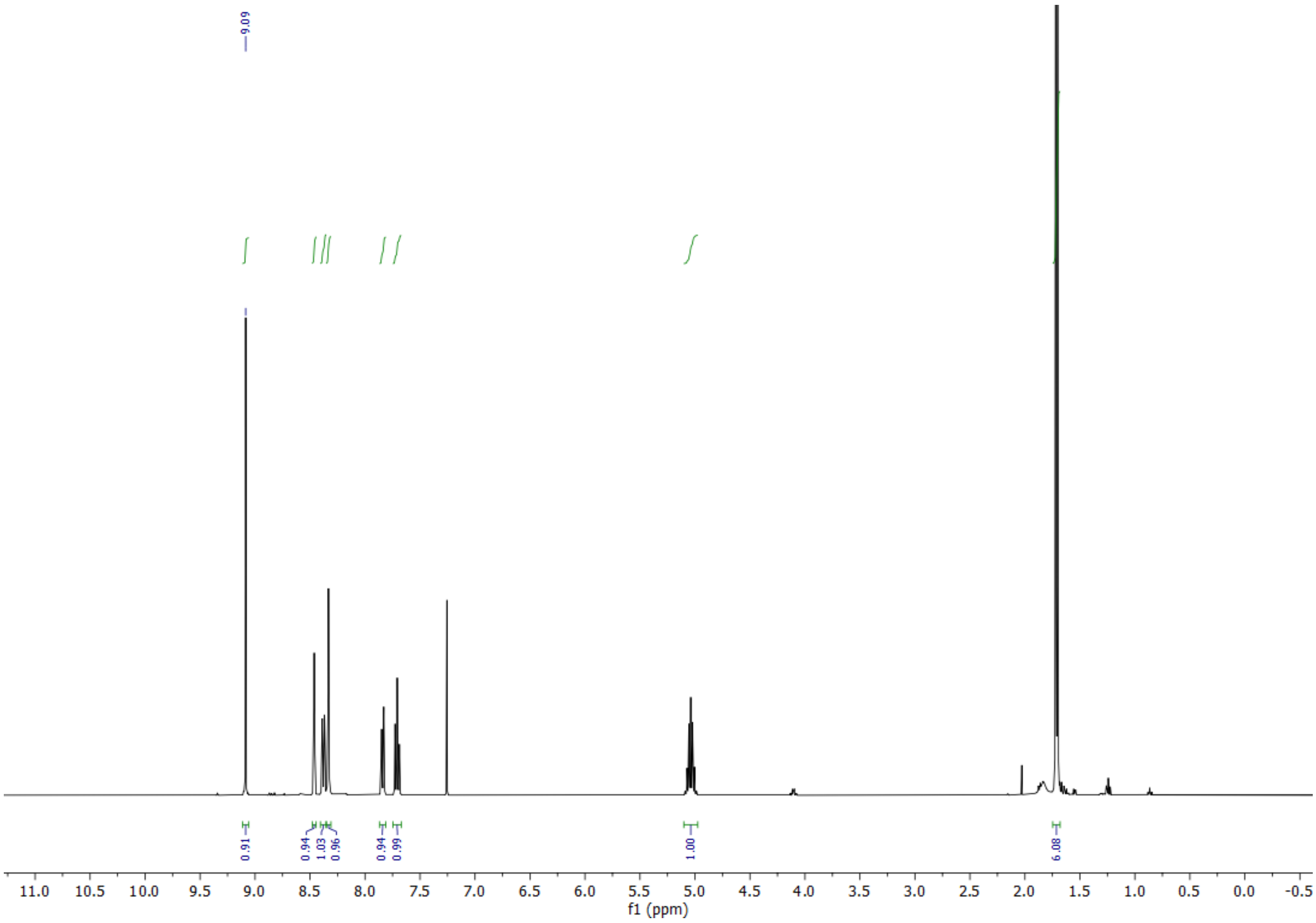
9.09
0.91
0.94
1.03
0.96
0.94
0.99
1.00
6.08
11.0 10.5 10.0 9.5 9.0 8.5 8.0 7.5 7.0 6.5 6.0 5.5 5.0 4.5 4.0 3.5 3.0 2.5 2.0 1.5 1.0 0.5 0.0 -0.5
f1 (ppm)

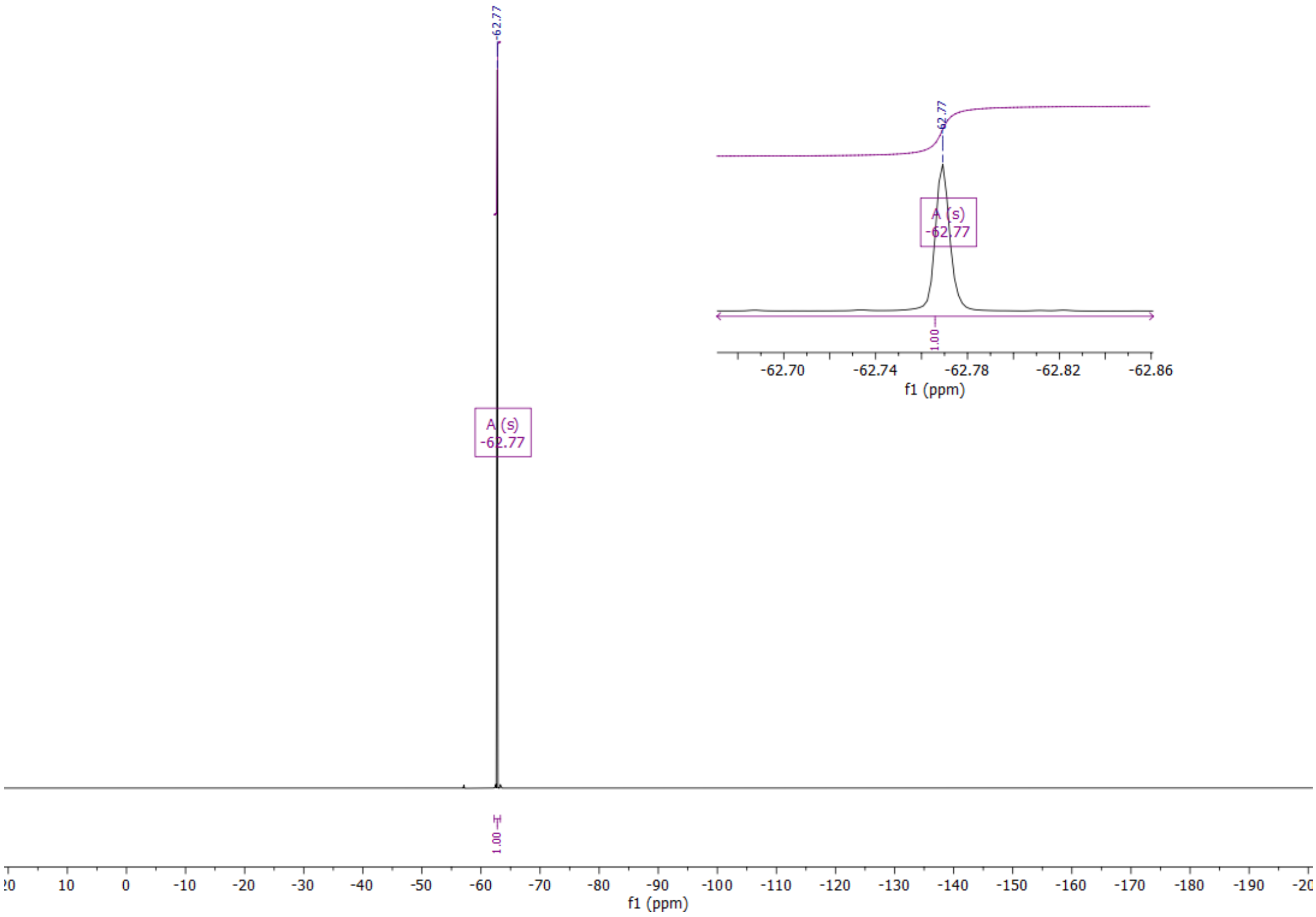
-62.77
A (s)
-62.77
1.00
10 0 -10 -20 -30 -40 -50 -60 -70 -80 -90 -100 -110 -120 -130 -140 -150 -160 -170 -180 -190
f1 (ppm)
-62.77
A (s)
-62.77
1.00
-62.70 -62.74 -62.78 -62.82 -62.86
f1 (ppm)

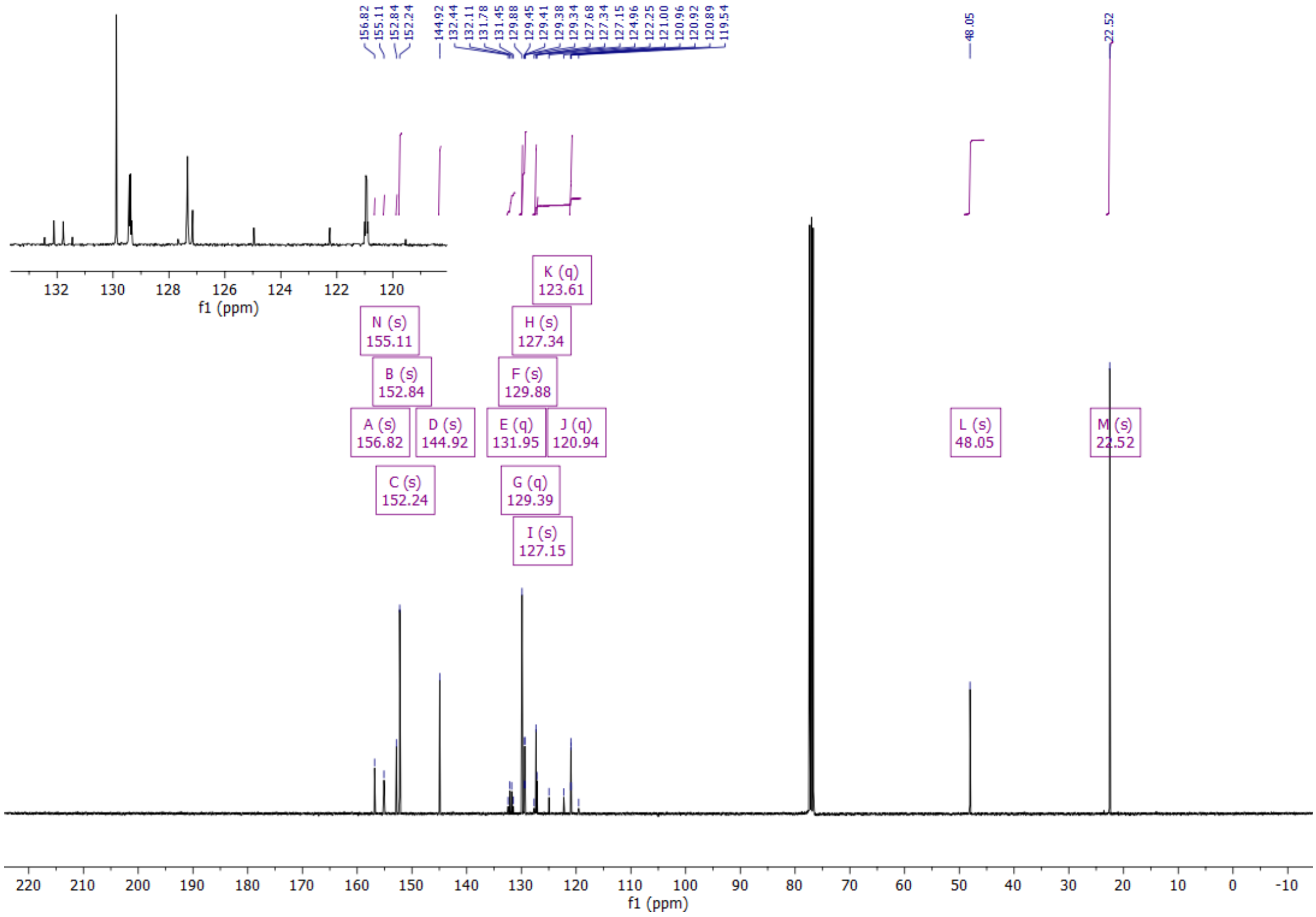

Fig. S7. ^{1}H, ^{19}F and ^{13}C NMR spectra of **4d**.

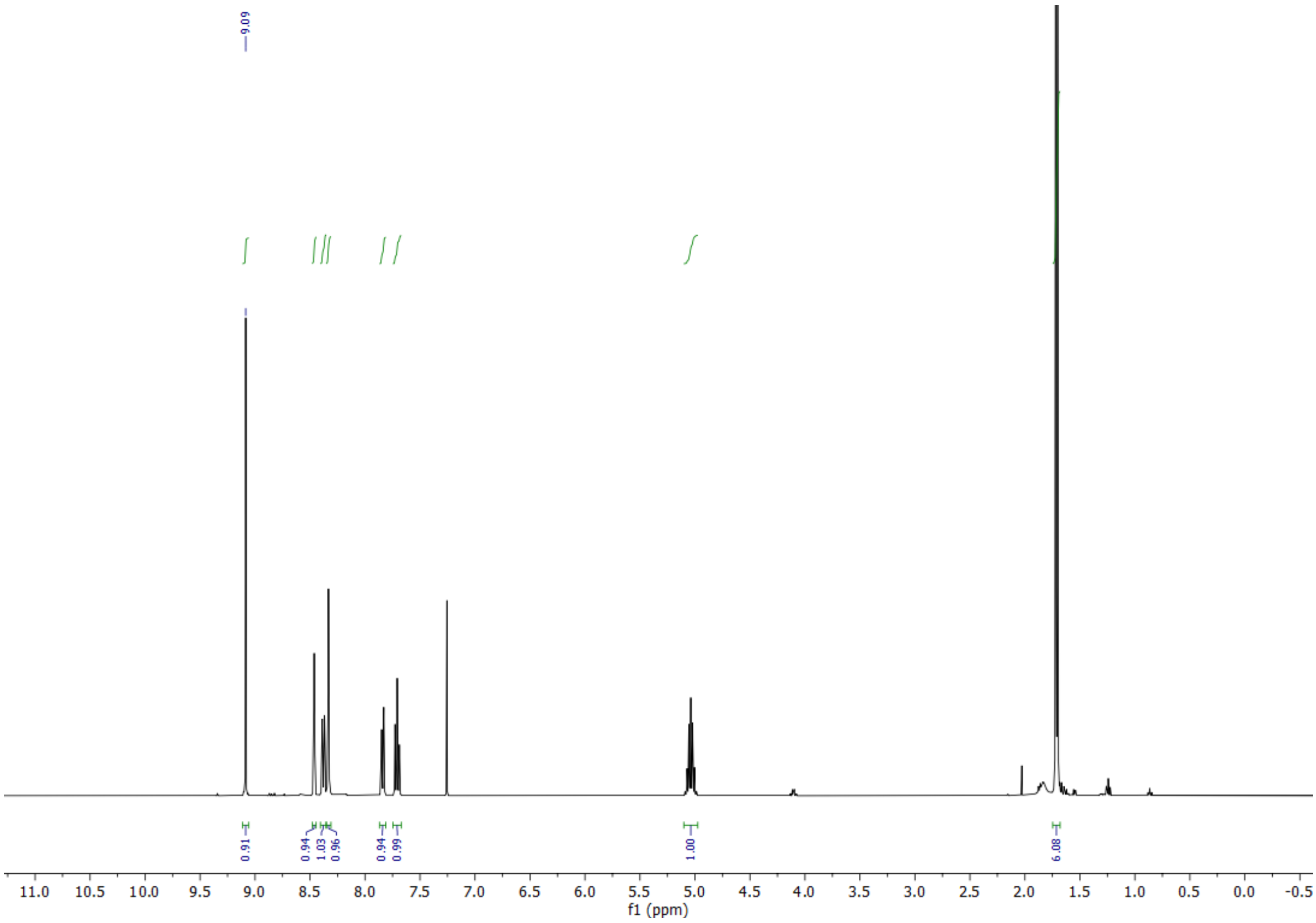

9.09
0.91
0.94
1.03
0.96
0.94
0.99
1.00
6.08
11.0 10.5 10.0 9.5 9.0 8.5 8.0 7.5 7.0 6.5 6.0 5.5 5.0 4.5 4.0 3.5 3.0 2.5 2.0 1.5 1.0 0.5 0.0 -0.5
f1 (ppm)

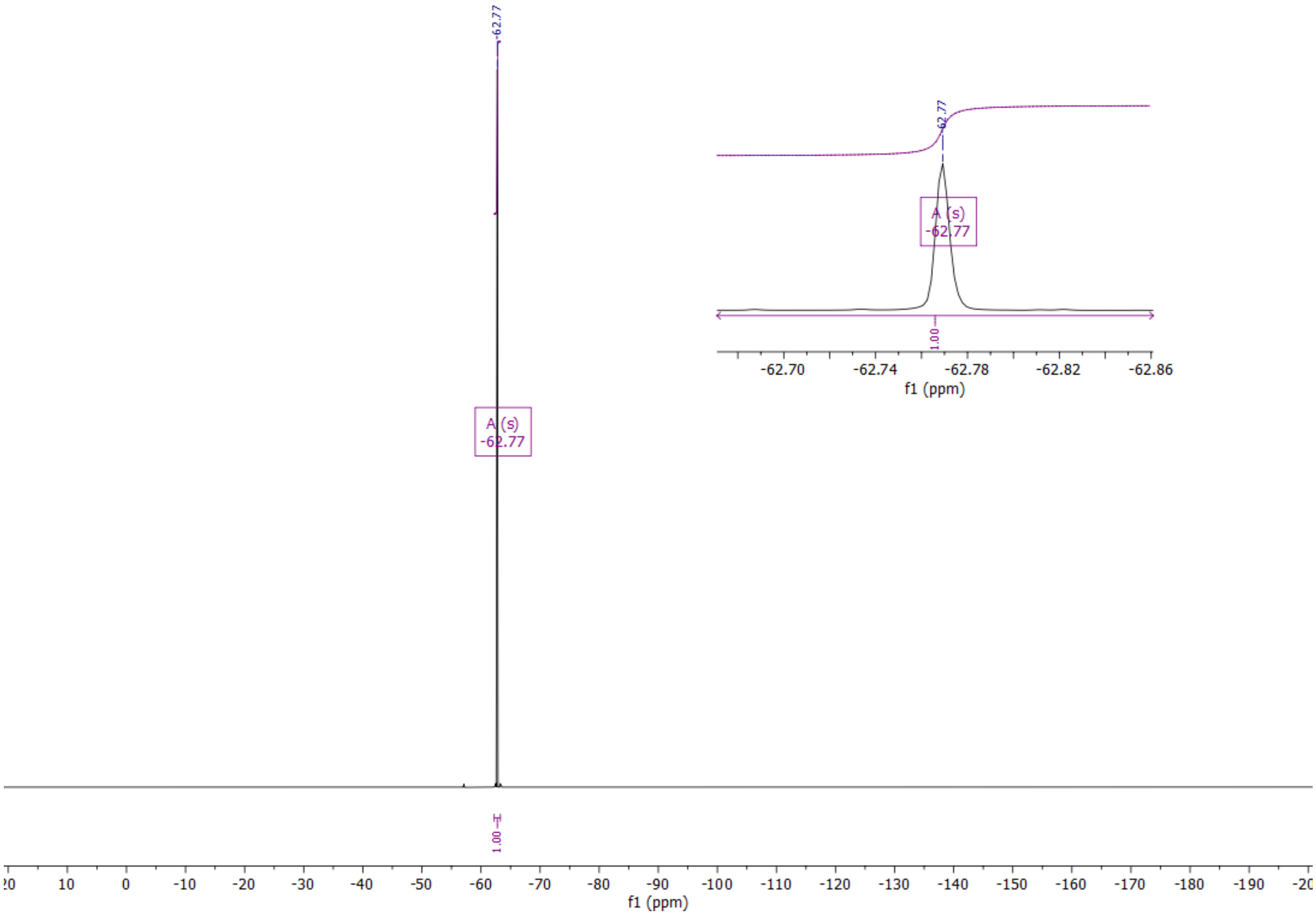

-62.77
A (s)
-62.77
1.00
-62.70 -62.74 -62.78 -62.82 -62.86
f1 (ppm)
10 0 -10 -20 -30 -40 -50 -60 -70 -80 -90 -100 -110 -120 -130 -140 -150 -160 -170 -180 -190
f1 (ppm)

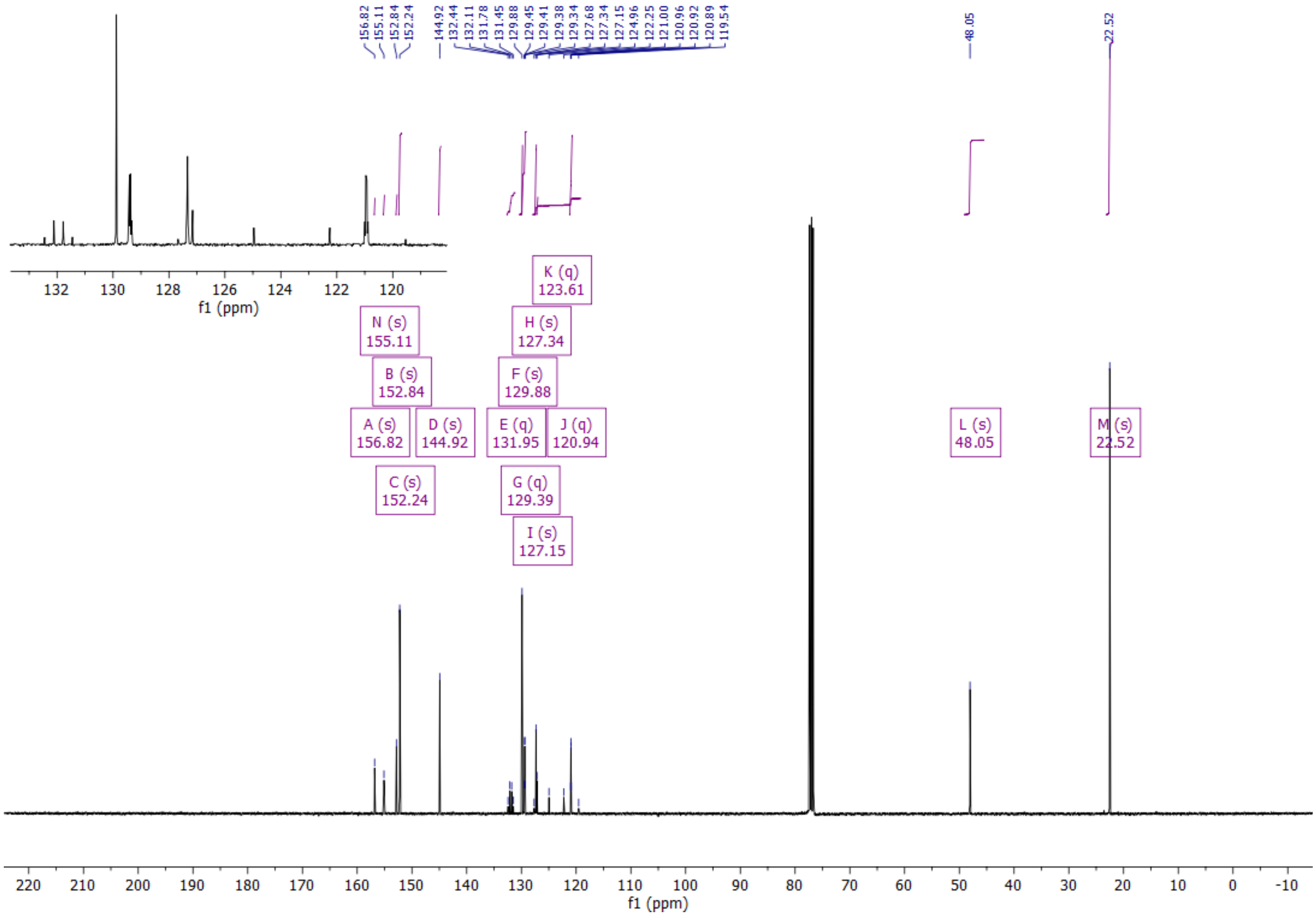

Fig. S8. ^{1}H, ^{19}F and ^{13}C NMR spectra of **4l**.

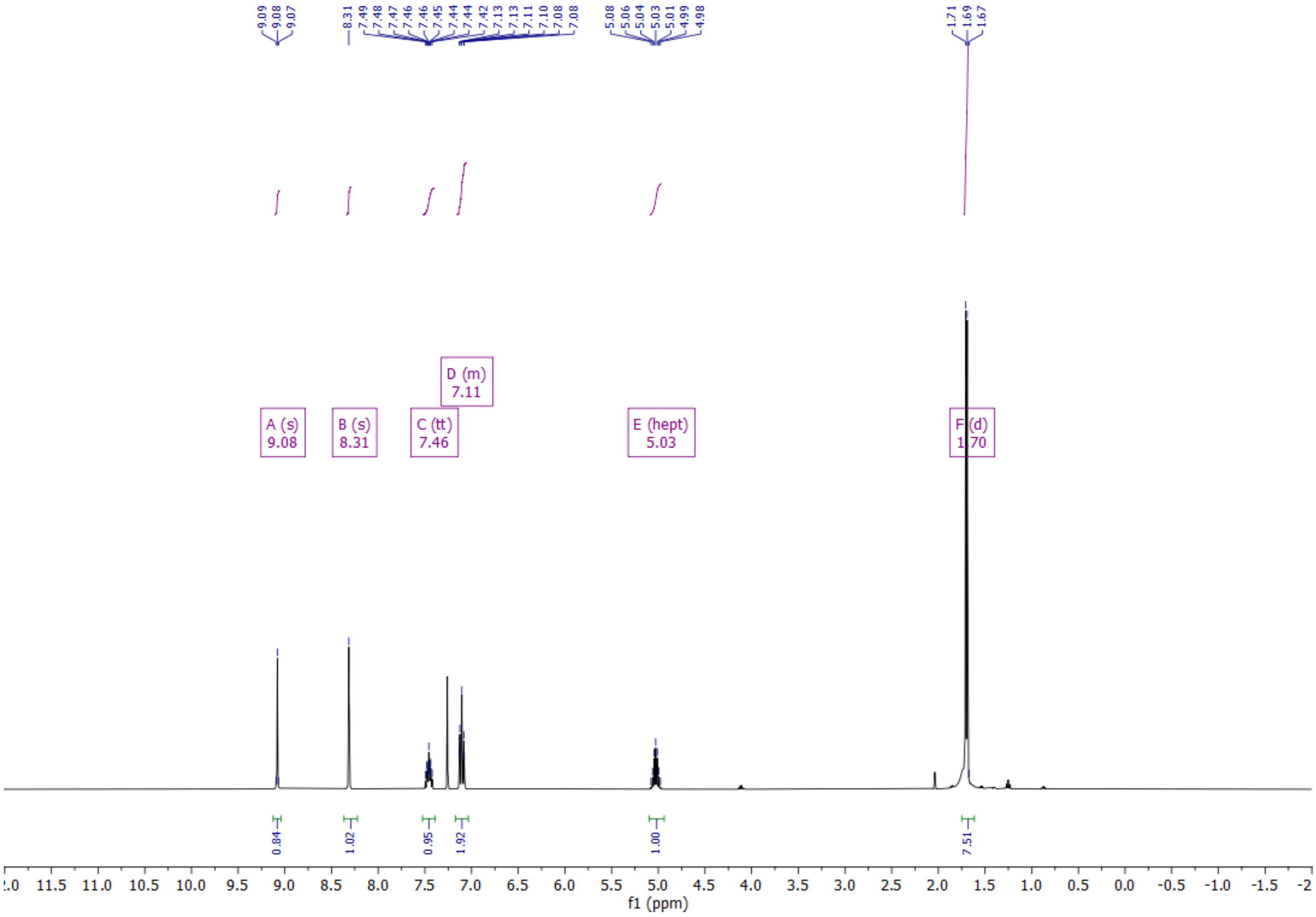
A (s)
9.08
B (s)
8.31
C (tt)
7.46
D (m)
7.11
E (hept)
5.03
F (d)
1.70
f1 (ppm)

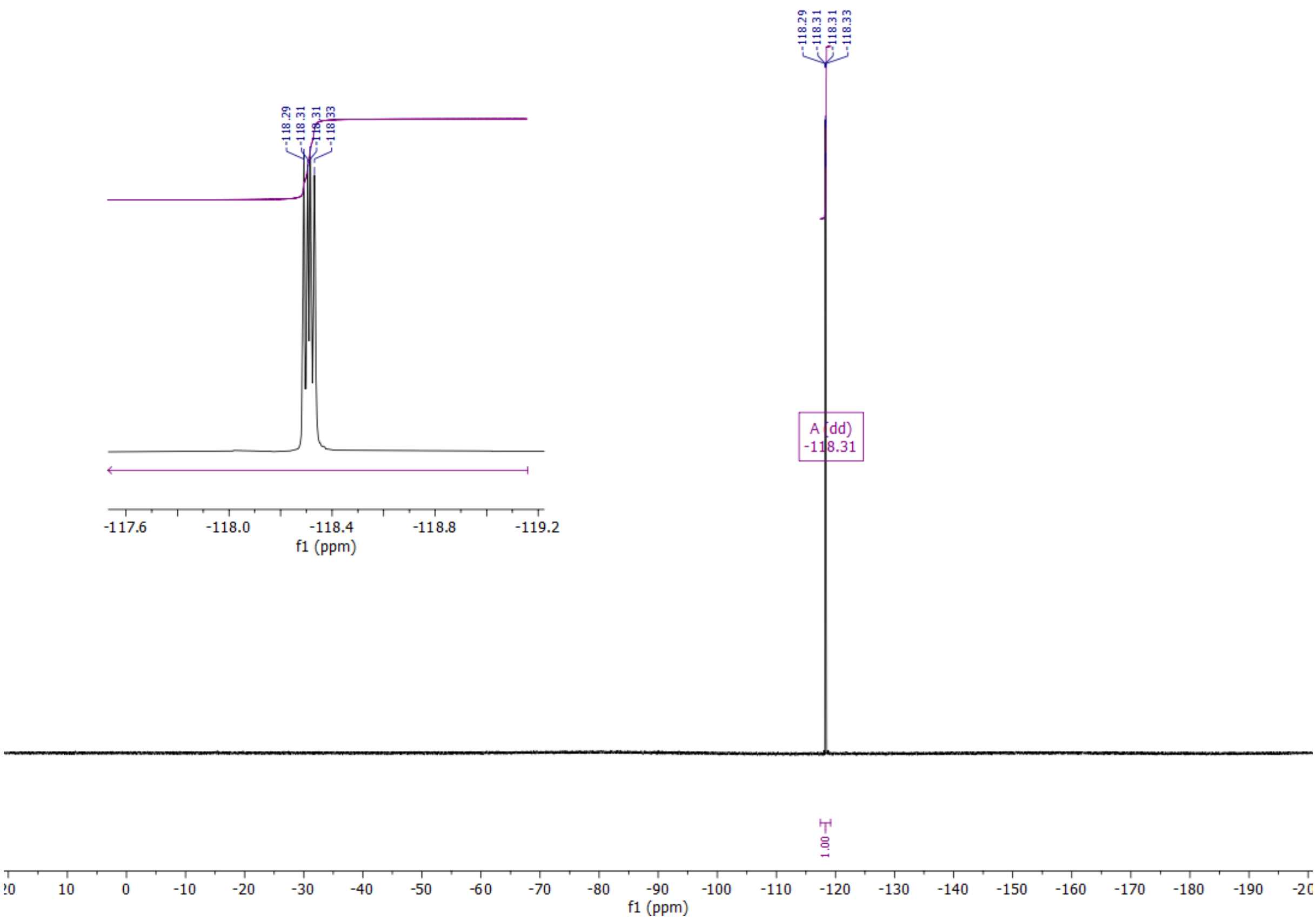
A (dd)
-118.31
f1 (ppm)

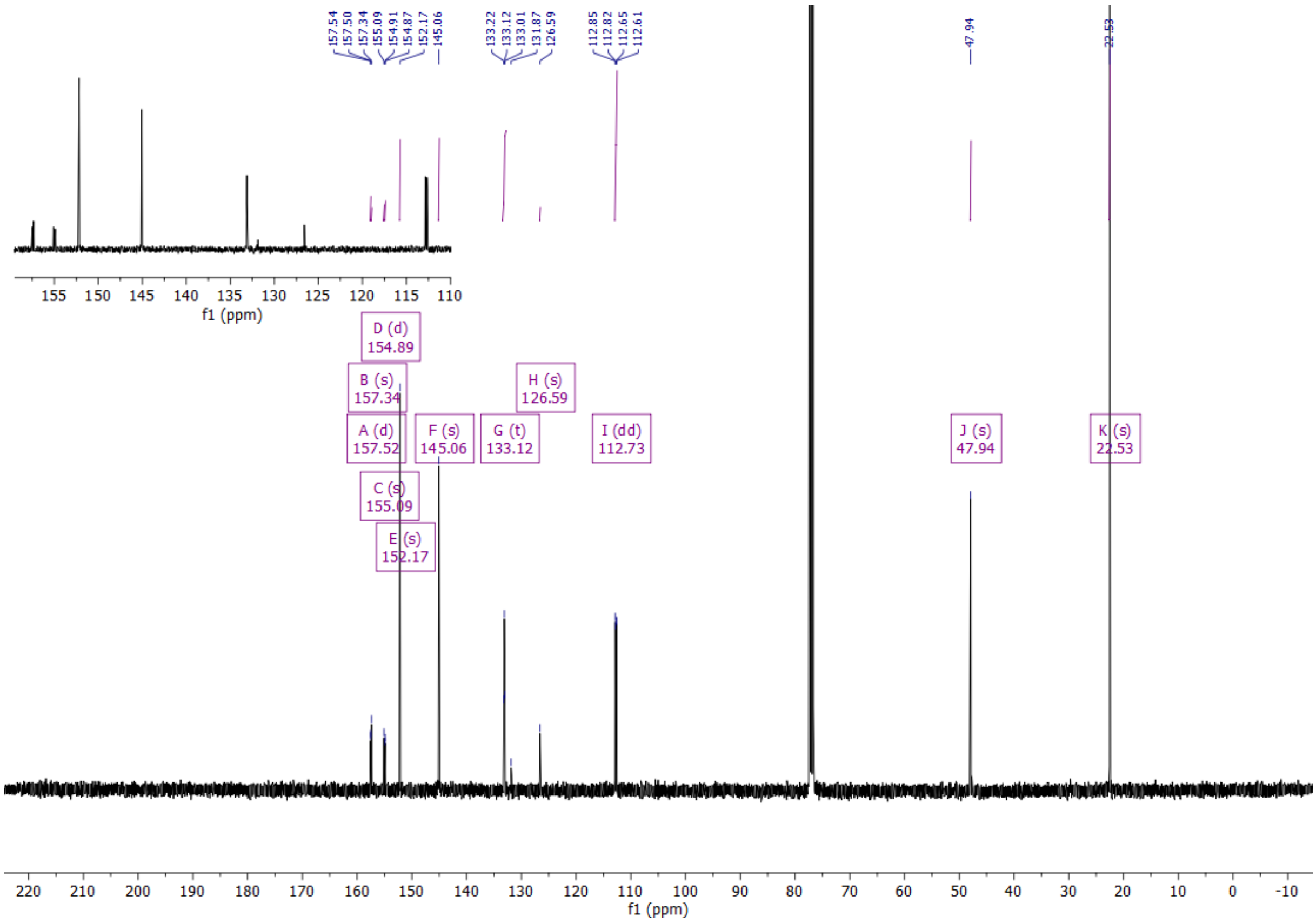

Fig. S9. ^{1}H, ^{19}F and ^{13}C NMR spectra of **4q**.

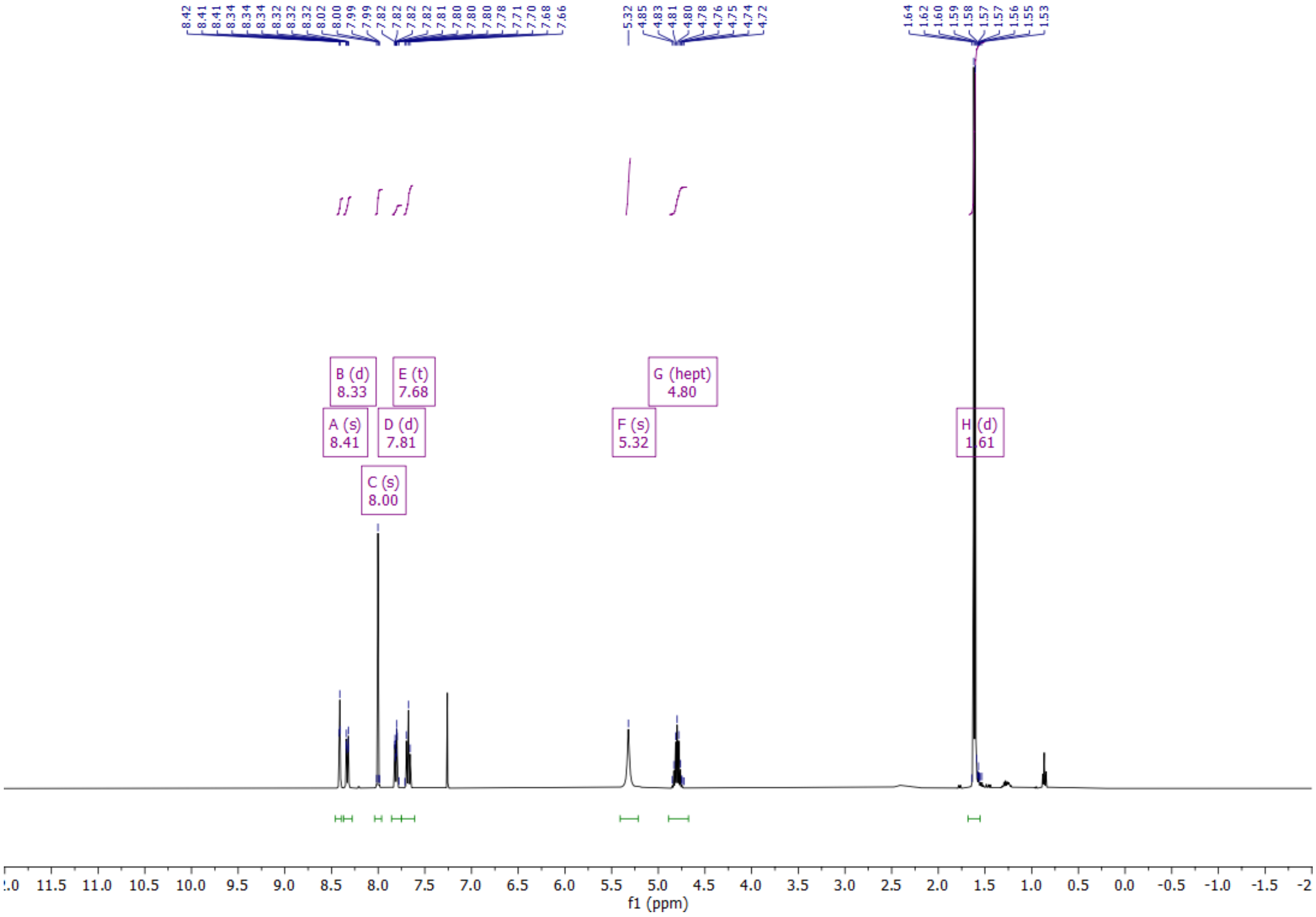
8.42 8.41 8.41 8.34 8.34 8.34 8.32 8.32 8.32 8.02 8.00 7.99 7.82 7.82 7.82 7.82 7.81 7.80 7.80 7.80 7.78 7.71 7.70 7.68 7.66
5.32 4.85 4.83 4.81 4.80 4.78 4.76 4.75 4.74 4.72
1.64 1.62 1.60 1.59 1.58 1.57 1.57 1.56 1.55 1.53
B (d) 8.33
E (t) 7.68
A (s) 8.41
D (d) 7.81
C (s) 8.00
G (hept) 4.80
F (s) 5.32
H (d) 1.61
f1 (ppm)

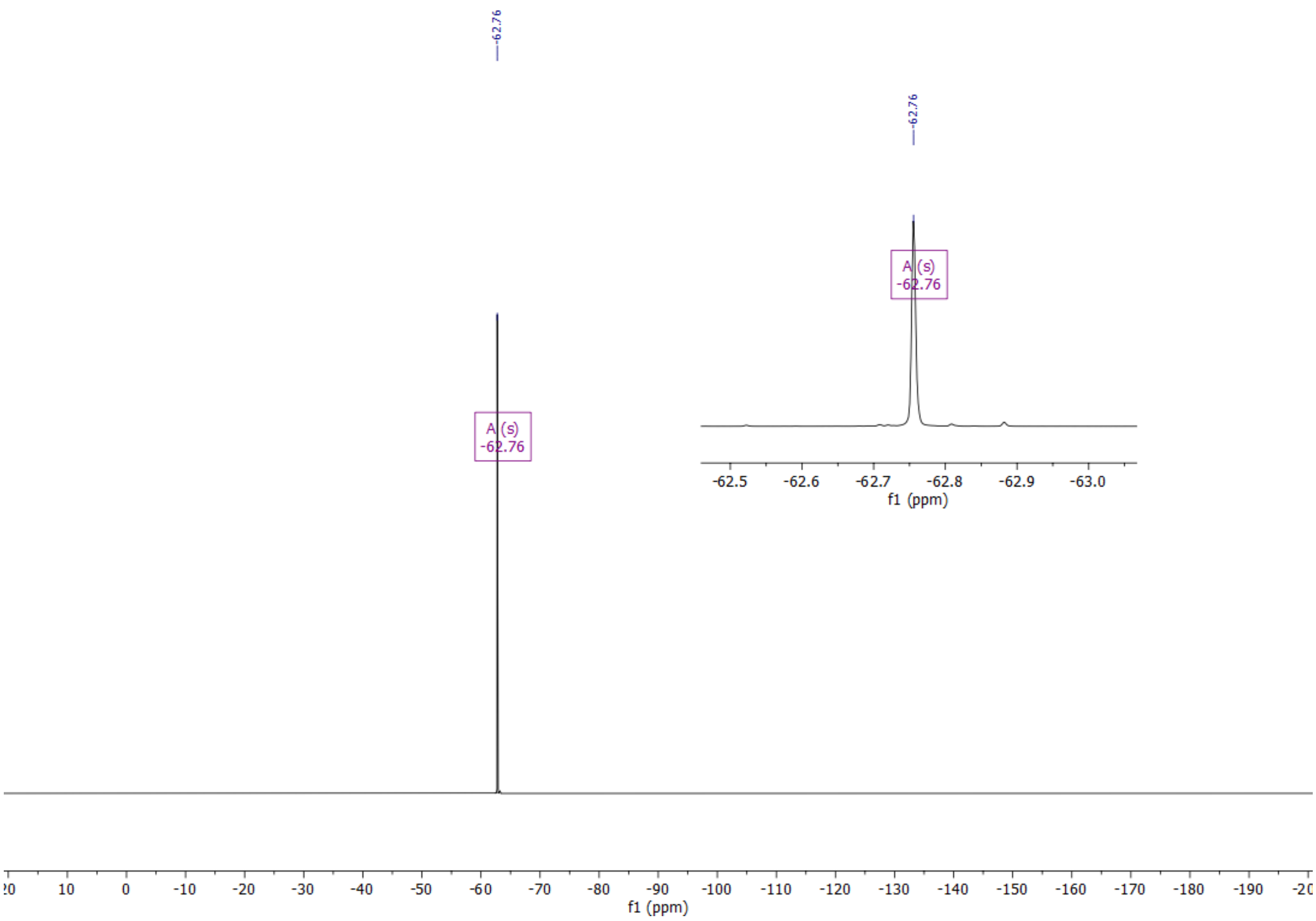
-62.76
A (s) -62.76
f1 (ppm)

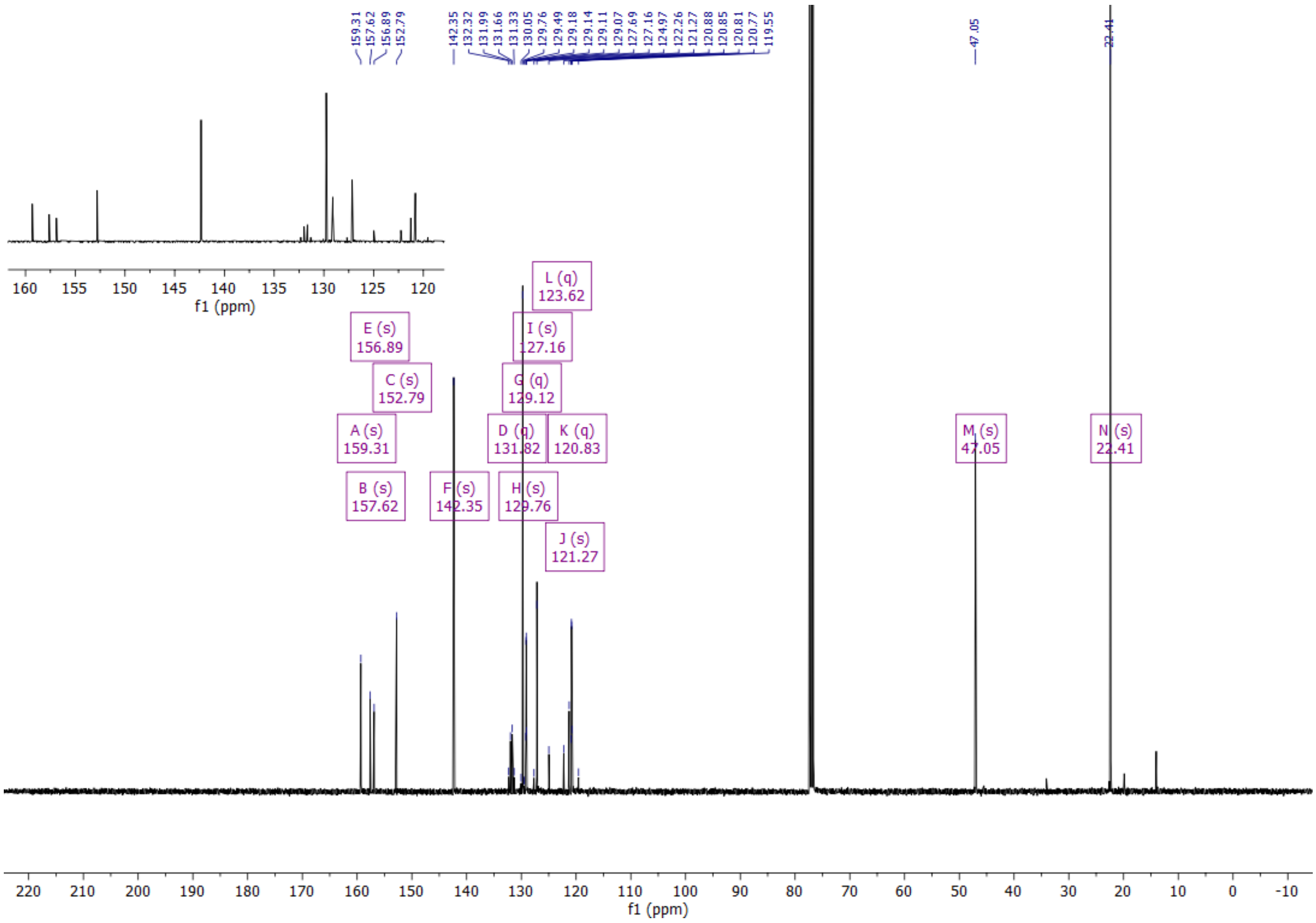

Fig. S10. ^{1}H, ^{19}F and ^{13}C NMR spectra of **5a**.

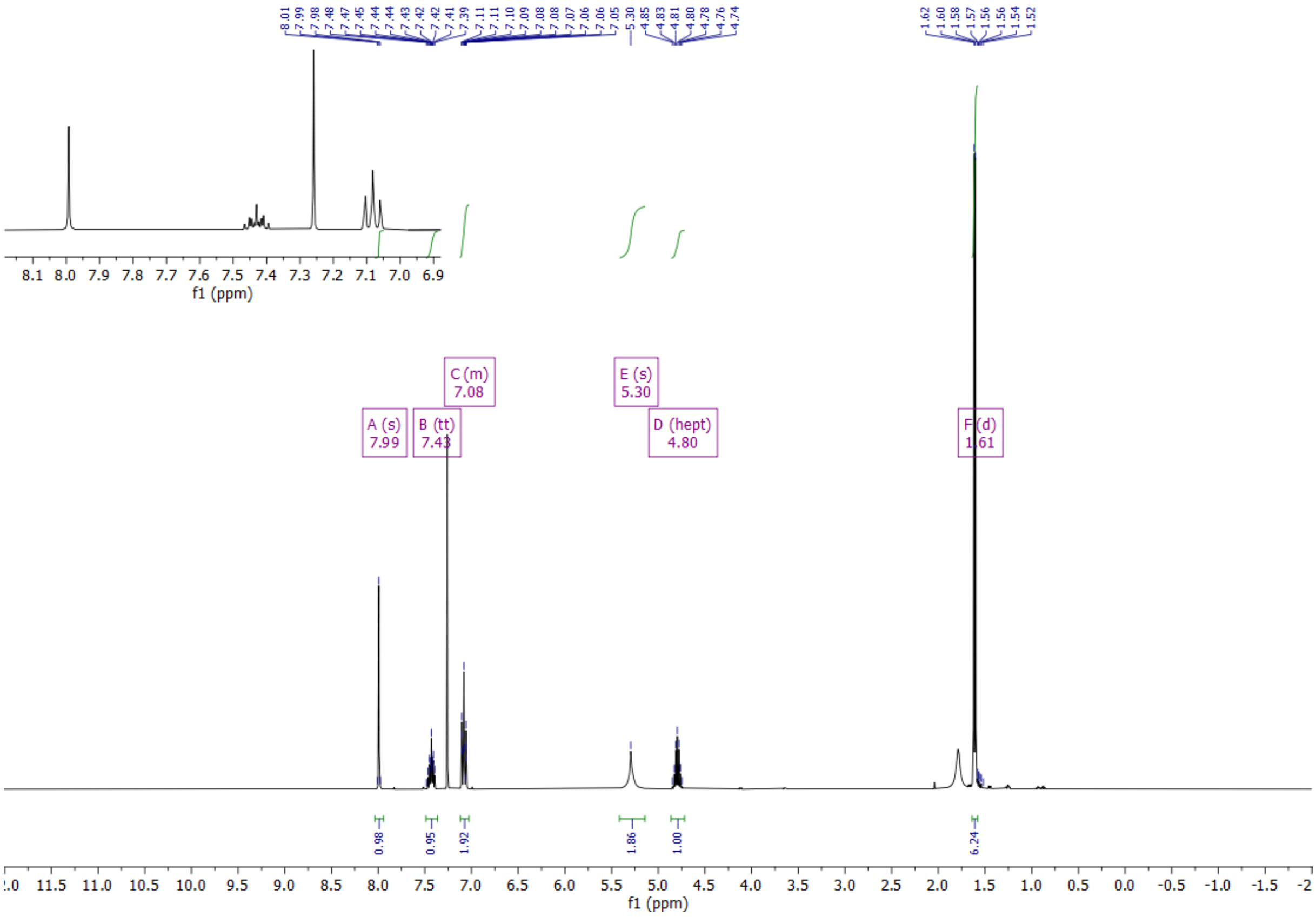
8.01
7.99
7.98
7.48
7.47
7.45
7.44
7.44
7.43
7.42
7.42
7.41
7.39
7.11
7.11
7.10
7.09
7.08
7.08
7.07
7.06
7.06
7.05
5.30
4.85
4.83
4.81
4.80
4.78
4.76
4.74
1.62
1.60
1.58
1.57
1.56
1.56
1.54
1.52
A (s) 7.99
B (tt) 7.43
C (m) 7.08
E (s) 5.30
D (hept) 4.80
F (d) 1.61
0.98
0.95
1.92
1.86
1.00
6.24
f1 (ppm)

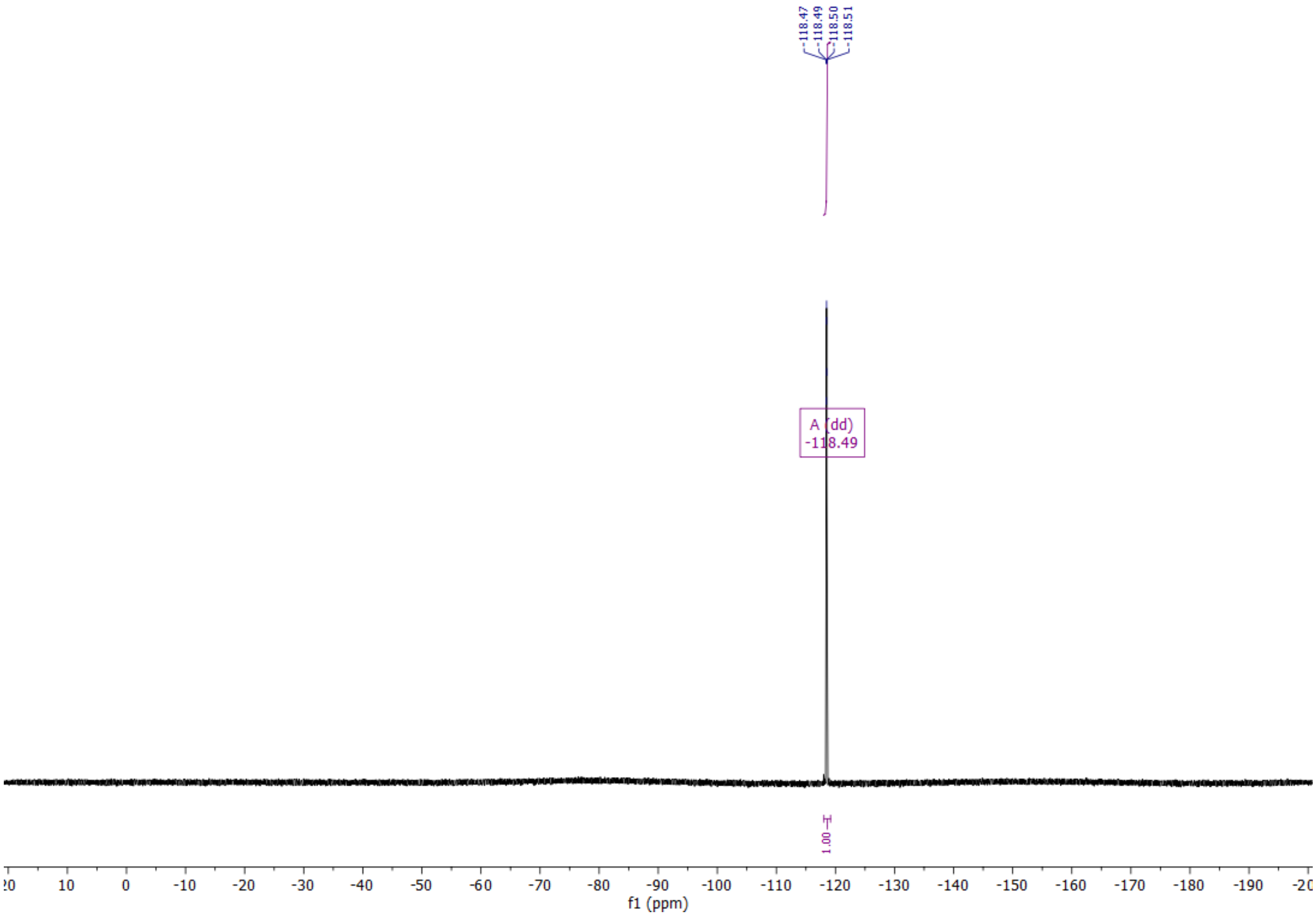
-118.47
-118.49
-118.50
-118.51
A (dd) -118.49
1.00
f1 (ppm)

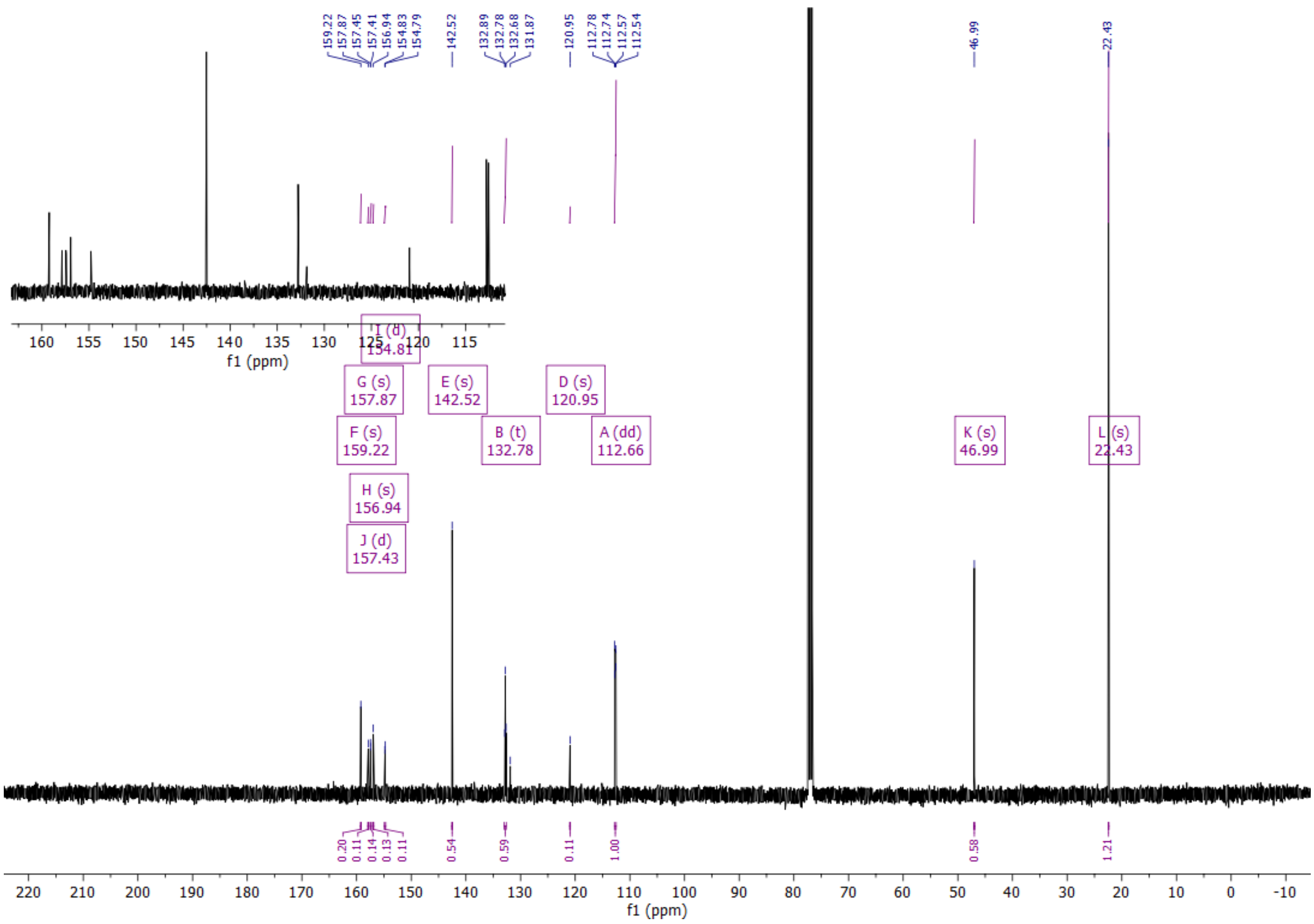

Fig. S11. ^{1}H, ^{19}F and ^{13}C NMR spectra of **5f**.

REFERENCES

[1] T. Hirota, W. G. Lewis, A. C. Liu, J. W. Lee, P. G. Schultz, and S. A. Kay, *Proc. Natl. Acad. Sci.* 2008, **105**, 20746-20751.

[2] D. Kolarski, W. Szymanski, and B. L. Feringa, *Org. Lett.* 2017, **19**, 5090-5093.
